# Multilayered regulation of autophagy by the Atg1 kinase orchestrates spatial and temporal control of autophagosome formation

**DOI:** 10.1016/j.molcel.2021.10.024

**Published:** 2021-12-16

**Authors:** Anne Schreiber, Ben C. Collins, Colin Davis, Radoslav I. Enchev, Angie Sedra, Rocco D’Antuono, Ruedi Aebersold, Matthias Peter

**Affiliations:** 1 Cellular Degradation Systems Laboratory, The Francis Crick Institute, 1 Midland Road, NW1 1AT London, UK; 2 Institute of Biochemistry, ETH Zürich, Otto-Stern-Weg 3, 8093 Zürich, Switzerland; 3 Institute of Molecular Systems Biology, ETH Zürich, Otto-Stern-Weg 3, 8093 Zürich, Switzerland; 4 School of Biological Sciences, Queen’s University of Belfast, 19 Chlorine Gardens, BT9 5DL Belfast, UK; 5 Crick Advanced Light Microscopy (CALM) STP, The Francis Crick Institute, 1 Midland Road, NW1 1AT London, UK; 6 Visual Biochemistry Laboratory, The Francis Crick Institute, 1 Midland Road, NW1 1AT London, UK

**Keywords:** autophagy, signaling, phosphorylation, protein kinases, protein phosphatases, ubiquitin-like proteins, Atg8 lipidation, metabolism

## Abstract

Autophagy is a conserved intracellular degradation pathway exerting various cytoprotective and homeostatic functions by using *de novo* double-membrane vesicle (autophagosome) formation to target a wide range of cytoplasmic material for vacuolar/lysosomal degradation. The Atg1 kinase is one of its key regulators, coordinating a complex signaling program to orchestrate autophagosome formation. Combining *in vitro* reconstitution and cell-based approaches, we demonstrate that Atg1 is activated by lipidated Atg8 (Atg8-PE), stimulating substrate phosphorylation along the growing autophagosomal membrane. Atg1-dependent phosphorylation of Atg13 triggers Atg1 complex dissociation, enabling rapid turnover of Atg1 complex subunits at the pre-autophagosomal structure (PAS). Moreover, Atg1 recruitment by Atg8-PE self-regulates Atg8-PE levels in the growing autophagosomal membrane by phosphorylating and thus inhibiting the Atg8-specific E2 and E3. Our work uncovers the molecular basis for positive and negative feedback imposed by Atg1 and how opposing phosphorylation and dephosphorylation events underlie the spatiotemporal regulation of autophagy.

## Highlights

• Lipidated Atg8 activates Atg1, stimulating substrate phosphorylation at phagophores
• Phosphorylation of Atg13 by Atg1 dissociates the Atg1 complex at the PAS
• Atg1 downregulates Atg8 lipidation at phagophores by inhibiting Atg3 and Atg12
• Protein phosphatase activity reverses the inhibitory functions of Atg1

## Graphical abstract

**Figure:**
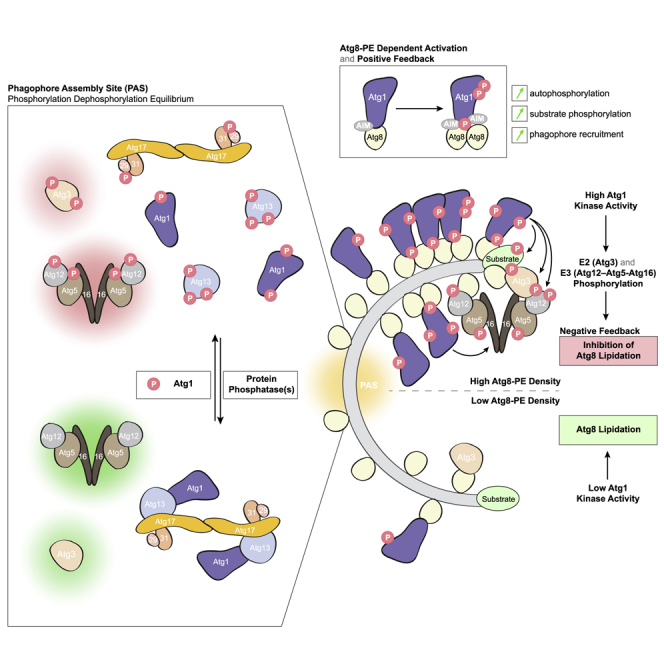

## Teaser

Schreiber et al. show that upon autophagy induction, lipidated Atg8 activates the Atg1 kinase. This downregulates Atg8 lipidation as Atg1 phosphorylates two of the enzymes involved in Atg8 lipidation, inhibiting their activity. Phosphorylation of Atg13 further dissociates the Atg1 complex, regulating Atg1 complex dynamics at the phagophore assembly site.

## Introduction

Macroautophagy, hereafter referred to as autophagy, is an intracellular degradation pathway that uses *de novo* double-membrane vesicle (autophagosome) formation to engulf cytoplasmic material. Degradation of the enclosed cellular material by fusion with the vacuole/lysosome allows recycling of cellular building blocks, explaining the function of autophagy in cellular homeostasis, particularly during nutrient starvation. While bulk autophagy mediates the seemingly random uptake of cytoplasmic material, selective autophagy pathways target potentially cytotoxic structures such as damaged organelles, protein aggregates, or invading pathogens in a highly discriminating manner. Deregulation of autophagy is involved in the etiology of diverse human diseases such as cancer, neurodegeneration, and infectious diseases (Dikic and Elazar, 2018). Hence, modulating this cellular self-defense pathway holds promise for treating some of the most prevalent human diseases and for slowing the cellular aging process.

The protein kinase autophagy-related 1 (Atg1) is one of the key regulators of autophagy. Under nutrient-rich conditions, Atg1 forms a complex with Atg13, promoting selective autophagy pathways (Kamber et al., 2015; Shintani and Klionsky, 2004; Torggler et al., 2016). Upon starvation, target of rapamycin complex 1 (TORC1) inactivation allows recruitment of the Atg17-Atg29-Atg31 subcomplex, triggering bulk autophagy (Kabeya et al., 2005, 2009; Kamada et al., 2000). The dimeric architecture of the Atg1 complex (Ragusa et al., 2012) and its arrangement into a higher-order mesh-like structure (Yamamoto et al., 2016) is thought to tether Atg9-containing vesicles, thus initiating autophagosomal membrane formation at the pre-autophagosomal structure (PAS) (Kishi-Itakura et al., 2014; Mari et al., 2010; Rao et al., 2016). Phosphatidylinositol 3-phosphate (PI3P) synthesis in the growing phagophore catalyzed by the Vps34^Atg14/Atg38^ complex recruits the Atg18-Atg2 complex (Obara et al., 2008), which in turn contributes to autophagosome formation by tethering membranes and transfering lipids (Kotani et al., 2018; Maeda et al., 2019; Osawa et al., 2019; Valverde et al., 2019) and by recruiting part of the Atg8 lipidation machinery (Dooley et al., 2014; Strong et al., 2021).

Covalent conjugation of the ubiquitin-like (Ubl) protein Atg8 to phosphatidylethanolamine (PE) is a hallmark of autophagy. Atg8 lipidation requires proteolytic processing by Atg4 and the action of an E1- (Atg7), E2- (Atg3), and E3-like enzyme (Atg12–Atg5-Atg16) (Hurley and Schulman, 2014). Atg8 lipidation regulates autophagosomal membrane formation (Kirisako et al., 1999; Nakatogawa et al., 2007) and phagophore association of several Atg proteins containing Atg8-interacting motifs (AIMs), including Atg1, Atg3, Atg4, and the Atg12–Atg5-Atg16 complex (Abreu et al., 2017; Kaufmann et al., 2014; Kraft et al., 2012; Nakatogawa et al., 2012b; Ngu et al., 2015; Suzuki et al., 2013; Yamaguchi et al., 2010). Moreover, AIM-dependent cargo adaptor binding by Atg8 promotes cargo sequestration during selective autophagy (Johansen and Lamark, 2020; Schreiber and Peter, 2014).

Despite the importance of autophagy for both health and disease, progress toward understanding autophagosome formation has been hindered in part by our limited understanding of how Atg1-mediated phosphorylation affects the core autophagy machinery, its catalytic activities, and protein-protein interactions. Atg1 kinase activity is essential for both bulk and selective autophagy (Kijanska et al., 2010; Matsuura et al., 1997; Yeh et al., 2010), and inactivation of Atg1 has been shown to prevent autophagosomal membrane formation (Suzuki et al., 2013). A number of Atg1/Ulk1 kinase targets have been reported, including Atg2, Atg4, Atg6, Atg9, Atg13, Atg16, Atg19, Atg23, Atg26, Atg29, Atg33, Atg34, FIP200, Vps15, and Vps34 (Alsaadi et al., 2019; Egan et al., 2015; Feng et al., 2015, 2016; Hu et al., 2019; Kamber et al., 2015; Mercer et al., 2021; Papinski et al., 2014; Pengo et al., 2017; Pfaffenwimmer et al., 2014; Rao et al., 2016; Russell et al., 2013; Sánchez-Wandelmer et al., 2017). Atg1-mediated phosphorylation is thought to primarily exert stimulatory functions. Accordingly, phosphorylation of Atg9 and the Vps34^Atg14/Atg38^ complex subunit Atg6 are both required for autophagy (Feng et al., 2016; Papinski et al., 2014; Russell et al., 2013). However, Atg1-mediated phosphorylation has also been shown to inhibit autophagy by downregulating Atg4 activity (Pengo et al., 2017; Sánchez-Wandelmer et al., 2017). Moreover, protein phosphatases (PPs) are required for autophagy; while PP2A and the two redundant PP2C-type PPs, Ptc2 and Ptc3, regulate bulk autophagy (Bánréti et al., 2012; Memisoglu et al., 2019; Ogura et al., 2010; Pengo et al., 2017; Tal et al., 2007; Wong et al., 2015; Yeasmin et al., 2016), both Ptc6 and Ptc2/Ptc3 have been shown to promote selective autophagy pathways (Memisoglu et al., 2019; Tal et al., 2007).

Here, we recombinantly expressed and purified the core autophagy machinery, and characterized enzymatic activities and protein-protein interactions directly regulated by Atg1 kinase activity. We show that Atg1 is recruited and activated by Atg8-PE. Surprisingly, Atg1 downregulates Atg8 lipidation by inhibiting the Atg8-specific E2 (Atg3) and E3 (Atg12–Atg5-Atg16), generating negative feedback that self-regulates Atg8-PE levels in the growing phagophore. Moreover, Atg1 kinase activity regulates its own assembly state by triggering the disassembly of Atg1-based complexes at the PAS. Our studies thus provide mechanistic insights into Atg1 activation, Atg1 complex dynamics, and the spatiotemporal regulation of Atg8 lipidation, highlighting the phagophore as a critical signaling platform.

## Results

### A recombinant autophagy system allows the identification of Atg1 targets

**Figure 1:**
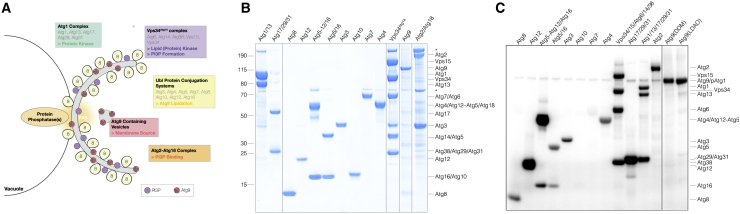
Reconstitution of the core autophagy machinery allows Atg1 substrate identification *in vitro* (A) Overview of the *S. cerevisiae* core autophagy machinery and its respective functions. Autophagosome formation is initiated at the PAS, requiring both Atg1 kinase and PP activity. (B) SDS-PAGE analysis of recombinant Atg proteins expressed and purified for this study. Asterisks, co-purifying insect cell proteins. (C) Autoradiograph of *in vitro* kinase assays depicting Atg1 substrates.

To mechanistically understand how Atg1 regulates autophagy, we recombinantly expressed and purified the *Saccharomyces cerevisiae* core autophagy machinery, including the Atg1-Atg13-Atg17-Atg29-Atg31 complex, the Vps34^Atg14/Atg38^ complex, Atg9, the Atg2-Atg18 complex, the two interconnected Ubl protein conjugation systems (Atg3, Atg7, Atg8, Atg10, Atg12, Atg12–Atg5-Atg16), and Atg4 (Figures 1A and 1B). Atg1 was expressed either alone or as part of the Atg1-Atg13 or Atg1-Atg13-Atg17-Atg29-Atg31 complex (Figures 1B and S1A), allowing us to directly study the functional impact of Atg1-mediated phosphorylation on the core autophagy machinery.

*In vitro* kinase assays confirmed that wild-type (WT) Atg1 readily phosphorylates itself (Figures 1C and S1B) and previously reported *in vivo* substrates, including Atg2, Atg4, Atg6, Atg9, Atg13, Atg29, and Atg31 (Figures 1C, S1B–S1G, and S1J). Recombinant Atg1 also phosphorylated substrates so far only observed in mammalian cells (Vps34 and Atg16) (Figures 1C, S1F, and S1G) and Atg proteins not previously described as Atg1 targets, including Atg18, Atg38, and several members of the Atg8 lipidation machinery such as Atg3, Atg12, Atg5, and Atg8 itself (Figures 1C, S1F–S1H, S1K, and S1L). In contrast, Atg7, Atg10, Atg14, and Atg17 were not significantly phosphorylated *in vitro* (Figures 1C, S1E, S1G, and S1J), suggesting that they are unlikely Atg1 targets.

To further analyze the specificity of our *in vitro* system, we compared the phosphorylation of a subset of Atg1 targets with two other protein kinases, Hrr25 and Tpk1 (Figures S1I–S1M), as both have previously been shown to phosphorylate Atg proteins (Stephan et al., 2009; Tanaka et al., 2014; Mochida et al., 2014; Pfaffenwimmer et al., 2014). As expected, Tpk1 also phosphorylated Atg13 (Figure S1J), while neither Tpk1 nor Hrr25 significantly phosphorylated Atg1 substrates involved in Atg8 lipidation (Figures S1K and S1L). These data suggest that our recombinant system provides a versatile and specific tool to rapidly screen for direct Atg1 substrates, in particular for Atg proteins whose phosphorylation is difficult to detect *in vivo* due to their inherently low expression levels (Ho et al., 2018).

### Atg8 lipidation stimulates both Atg1 autophosphorylation and Atg1-mediated substrate phosphorylation

**Figure 2:**
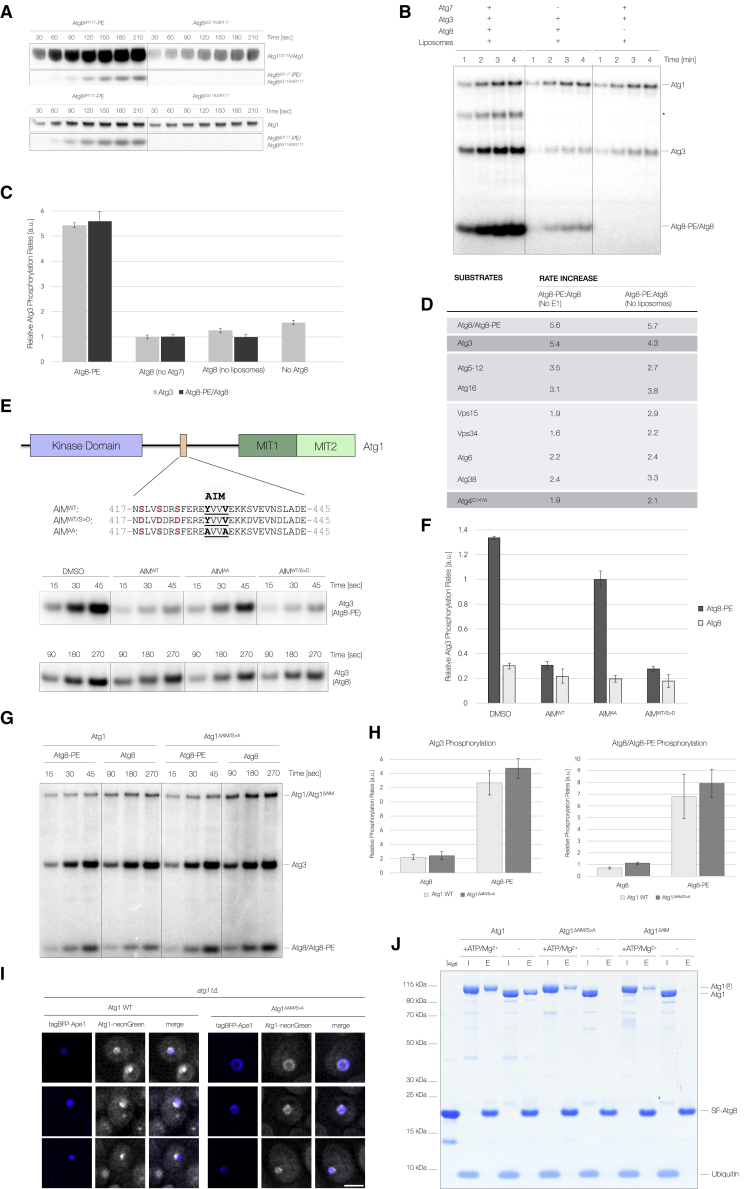
Atg8-PE stimulates Atg1 kinase activity (A) Atg1-mediated phosphorylation of catalytically inactive Atg1^D211A^ was monitored in the presence of either 4 μM lipidated Atg8^ΔR117^ (Atg8^ΔR117^-PE) or non-lipidated Atg8^ΔG116/ΔR117^ (upper panel). Before the addition of Atg1, Atg8^ΔR117^ and Atg8^ΔG116/ΔR117^ were incubated with Atg7, Atg3, and liposomes (55% PE, 35% phosphatidylcholine [PC], and 10% phosphatidylinositol [PI]) to promote Atg8^ΔR117^ lipidation. Atg1^D211A^ (2.5 μM) was added, and *in vitro* kinase assays were started by the addition of WT Atg1 (50 nM). Control reactions (lower panel) did not contain Atg1^D211A^ reporting only on Atg1 autophosphorylation. Time points were analyzed by SDS-PAGE and autoradiography. (B) Atg1-dependent phosphorylation of Atg8 and Atg3 was monitored in a time-dependent manner in the presence of either Atg8 (Atg8^ΔR117^) or Atg8-PE (Atg8^ΔR117^-PE). Atg8 was lipidated before starting the *in vitro* kinase assays by addition of pre-phosphorylated Atg1. Control reactions lacked either Atg7 or Atg8. Time points were analyzed by SDS-PAGE and autoradiography. Asterisk, Atg8-Atg3 conjugate. (C) Relative quantification of Atg1-dependent Atg3 and Atg8/Atg8-PE phosphorylation rates (n = 3) in the presence of Atg8 (by omitting either Atg7 or liposomes) or Atg8-PE. Atg3 phosphorylation rates were also quantified in the absence of Atg8. Representative autoradiographs used for quantification are shown in Figure S2A. (D) Relative rate increase of Atg1-dependent substrate phosphorylation in the presence of Atg8-PE. Atg8-PE-dependent phosphorylation rates were compared to control reactions devoid of Atg7 or liposomes. Relative phosphorylation rates for the indicated Atg proteins were determined using Atg8, Atg3, Atg12–Atg5-Atg16, Vps34^Atg14/Atg38^, and Atg4^C147A^ as substrates. Atg1 was phosphorylated before substrate addition. (E) Schematic representation of *S. cerevisiae* Atg1 highlighting the Atg8-interacting motif (AIM) (salmon), kinase, and MIT domains (purple and green, respectively). Atg1 autophosphorylation sites and corresponding phosphomimicking mutations are shown in red. Atg1-dependent phosphorylation of Atg3 was assayed in the presence of WT (AIM^WT^) or mutated AIM peptides (AIM^AA^ and AIM^WT/S^^>^^D^). Competition experiments were carried out in the presence of either Atg8 (bottom row) or Atg8-PE (top row). Longer time points were used to monitor Atg3 phosphorylation in the presence of Atg8. (F) Quantification of the relative Atg1-dependent Atg3 phosphorylation rates in arbitrary units (a.u.) in the presence of the indicated AIM peptides and either Atg8 or Atg8-PE. (G) Phosphorylation of Atg3 and Atg8/Atg8-PE was compared between WT Atg1 and Atg1^ΔAIM/S^^>^^A^ (Atg1^S418A/S421A/S424A/Y429A/V432A^) in the presence of either Atg8 or Atg8-PE. Different time points are shown for Atg8 and Atg8-PE-containing reactions. (H) Quantification of the relative Atg1 and Atg1^ΔAIM/S^^>^^A^-dependent phosphorylation rates for Atg3 and Atg8/Atg8-PE in the presence of either Atg8 or Atg8-PE (n = 3). (I) Localization of neonGreen-tagged Atg1 and Atg1^ΔAIM/S^^>^^A^ in nitrogen-starved *atg11Δ* cells overexpressing tagBFP-Ape1. A single z stack is shown. Scale bar, 2 μm. (J) Atg1, Atg1^ΔAIM^ (Atg1^Y429A/V432A^), and Atg1^ΔAIM/S^^>^^A^ pull-down experiments using SF-tagged Atg8 as bait (I_Atg8_) were analyzed by SDS-PAGE. Atg1 preparations were either autophosphorylated (ATP/Mg^2+^) or dephosphorylated using λ-PP (−). To avoid differential Atg8 phosphorylation, all Atg1 preparations were treated with apyrase before addition to Atg8-coated FLAG resin. Ubiquitin was used as a specificity control.

As Atg8 emerged as an Atg1 target (Figures 1C, S1F, and S1L), we tested whether Atg8 conjugation to PE affects its Atg1-dependent phosphorylation. Strikingly, upon lipidation, both Atg1 autophosphorylation (Figure 2A) and Atg1-mediated phosphorylation of Atg8-PE increased drastically (Figures 2A–2C, S2A, and S2B). Atg8-PE also stimulated the Atg1-dependent phosphorylation of Atg3 (Figures 2B, 2C, S2A, and S2B) and the phosphorylation of a wide range of other Atg1 substrates (Figures 2D and S2C–S2E), suggesting a general effect for Atg8-PE in stimulating Atg1 substrate phosphorylation.

Atg1 directly binds Atg8 via an AIM (Kraft et al., 2012; Nakatogawa et al., 2012b), which is thought to direct Atg1 to growing phagophores (Suzuki et al., 2013). Mass spectrometry analysis of autophosphorylated Atg1 identified three phosphorylation sites N-terminal to this AIM (Figure 2E; Table S1), and this region was also phosphorylated *in vivo* (Hu et al., 2019; Lanz et al., 2021). However, mutating these phosphorylation sites did not aggrevate the bulk autophagy defect of an Atg1 AIM mutant (Figure S2F). To understand whether the Atg1 AIM is required for Atg8-PE-dependent stimulation of substrate phosphorylation, we carried out competition assays monitoring Atg1-dependent substrate phosphorylation in the presence of WT and phosphomimicking AIM peptides (Figure 2E). Interestingly, Atg3 phosphorylation was strongly impaired by either AIM peptide (Figures 2E and 2F), implying that AIM-dependent Atg8-PE binding is required to stimulate Atg1 kinase activity.

To corroborate this finding, we compared Atg8 and Atg8-PE-dependent substrate phosphorylation by Atg1 to an Atg1 AIM mutant with the three proceeding phosphorylation sites mutated to alanine (Atg1^ΔAIM/S^^>^^A^). Surprisingly, the Atg1^ΔAIM/S^^>^^A^ mutant had no defect when analyzing Atg3 and Atg8-PE phosphorylation in the presence of Atg8-PE (Figures 2G and 2H).

To understand whether Atg1^ΔAIM/S^^>^^A^ lost its ability to bind Atg8, we studied the phagophore localization of Atg1^ΔAIM/S^^>^^A^ in *atg11Δ* cells to exclude Atg8-independent recruitment pathways (Shintani and Klionsky, 2004; Suzuki et al., 2013; Schütter et al., 2020). Atg1^ΔAIM/S^^>^^A^ still localized to giant Ape1 structures in these cells (Figure 2I), suggesting the Atg8-dependent recruitment of Atg1^ΔAIM/S^^>^^A^.

To further explain these findings, we tested Atg8 binding *in vitro*. While Atg1 AIM mutants were unable to interact with Atg8 (Figure 2J), autophosphorylation surprisingly restored binding to Atg8 (Figures 2J, S2G and S2H) or Atg8-PE-containing liposomes (Figure S2I). In line with an autophosphorylation-dependent interaction, a catalytically inactive Atg1 AIM mutant (Atg1^ΔAIM/S^^>^^A_D211A^) was unable to bind Atg8 in the presence of ATP/Mg^2+^ (Figure S2G). Since Atg1^ΔAIM/S^^>^^A^ autophosphorylation stimulated binding to both phosphorylated and non-phosphoryaled Atg8 (Figures 2J and S2H), we conclude that Atg1 autophosphorylation enhances Atg8 binding by exposing at least one additional phosphorylation-regulated AIM.

To map its location, we expressed and purified different Atg1^ΔAIM/S^^>^^A^ truncation mutants (Figure S2J). While the largely disordered central region harbored no additional AIM (Figure S2K), both the N-terminal kinase and the C-terminal microtubule-interacting and transport (MIT) domains weakly bound Atg8. However, as Atg8 binding did not significantly increase upon autophosphorylation (Figures S2K and S2L) and no linear AIM could be identified so far, a more complex mechanism involving both kinase and MIT domains is conceivable.

We conclude, therefore, that autophosphorylation exposes an additional phosphorylation-regulated AIM in Atg1 and that Atg8-PE dependent recruitment of Atg1 upregulates its kinase activity, stimulating both Atg1 autophosphorylation and phagophore-associated substrate phosphorylation.

### Atg1-mediated phosphorylation of Atg13 dissociates the Atg1 complex

**Figure 3:**
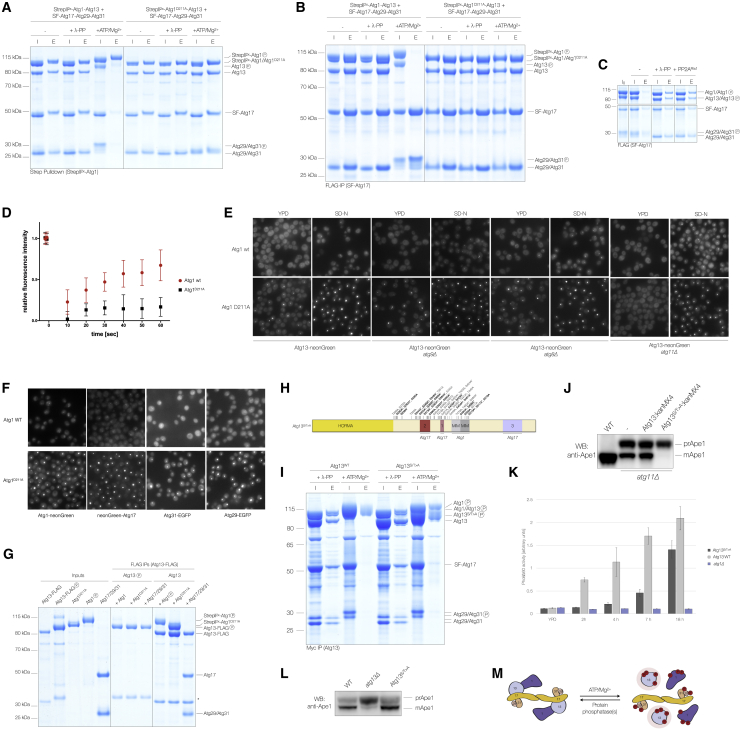
Atg1-mediated phosphorylation of Atg13 dissociates the Atg1 complex (A) StrepII^2x^-Atg1-Atg13 or catalytically inactive StrepII^2x^-Atg1^D211A^-Atg13 was mixed with Atg17-Atg29-Atg31 (input: I) and incubated with ATP/Mg^2+^ or λ-PP. Atg1 was immobilized using StrepTactin resin. Atg1 and co-purifying proteins were eluted (E) and analyzed by SDS-PAGE. (B) Atg1-Atg13 or Atg1^D211A^-Atg13 was mixed with SF-Atg17-Atg29-Atg31 (input: I) and incubated with or without ATP/Mg^2+^ or λ-PP. Atg17 and co-purifying proteins were eluted (E) and analyzed by SDS-PAGE. (C) The Atg1-Atg13-Atg17-Atg29-Atg31 complex was incubated with ATP/Mg^2+^ to trigger complex dissociation (I_0_). ATP was depleted using apyrase and samples were incubated with either λ-PP, PP2A^Rts1^, or no PP (I). SF-Atg17 was immobilized and pull-down elutions (E) were analyzed by SDS-PAGE. (D) Fluorescence recovery after photobleaching (FRAP) experiments monitoring Atg13-neonGreen in *atg11Δ* cells expressing either WT Atg1 or Atg1^D211A^. Quantification shows the relative fluorescence intensities after bleaching the Atg13-neonGreen signal as a function of time. (E) Fluorescence microscopy analysis of WT, *atg8Δ*, *atg9Δ*, or *atg11Δ* cells expressing Atg13-neonGreen in the presence of WT Atg1 or Atg1^D211A^. Cells were exponentially grown in nutrient-rich YPD medium or nitrogen starved for 4 h. Scale bar, 5 μm. (F) Fluorescence microscopy images of nitrogen-starved yeast expressing Atg1-neonGreen, Atg1^D211A^-neonGreen, or neonGreen-Atg17, Atg29-EGFP, or Atg31-EGFP in WT or *atg1*^*D211A*^ cells. Scale bar, 5 μm. (G) Atg13 was phosphorylated using substoichiometric amounts of Atg1. ATP was depleted using apyrase and phosphorylated, and non-phosphorylated Atg13 was immobilized. Autophosphorylated Atg1, Atg1^D211A^, or Atg17-Atg29-Atg31 were added, and Atg13 and co-purifying proteins were eluted and analyzed by SDS-PAGE. Asterisk, Atg13 truncation. (H) Serines and threonines mutated to alanine in the Atg13^S/T>A^ mutant are shown with respect to the N-terminal HORMA domain, Atg17 binding sites, and MIT-interacting motifs (MIMs). *In vivo* phosphorylation sites are marked by asterisks with putative Atg1-dependent phosphorylation sites underlined. Serines highlighted in black are phosphorylated by recombinant Atg1 *in vitro*. (I) Atg13^WT^ or Atg13^S/T>A^ were mixed with Atg1 and Atg17-Atg29-Atg31 (I) and incubated with either ATP/Mg^2+^ or λ-PP. Atg13 and Atg13^S/T>A^ were immobilized and elutions (E) were analyzed by SDS-PAGE. (J) WT and *atg11Δ* cells expressing either Atg13^WT^ or Atg13^S/T>A^ were nitrogen starved (7 h), and processing of precursor Ape1 (prApe1) to its mature form (mApe1) was monitored by western blotting (WB). (K) Pho8Δ60 assay measuring bulk autophagy in *atg1Δ* strains and yeast expressing either Atg13^WT^ or Atg13^S/T^^>^^A^. Cells were either exponentially grown in YPD medium or nitrogen starved for 2, 4, 7, or 18 h. Alkaline phosphatase activity was measured (n = 3) and plotted as relative Pho8Δ60 activity with standard deviation. (L) The indicated yeast strains were exponentially grown in YPD medium and Cvt pathway-dependent Ape1 processing was monitored by WB. (M) Model summarizing phosphorylation-dependent dissociation of the Atg1 complex.

While testing phosphorylation-dependent interactions of the Atg1 complex, we noticed that in the presence of ATP/Mg^2+^, Atg1 mainly bound to Atg8, while Atg13 and Atg17-Atg29-Atg31 were largely lost (Figure S3A). This was unexpected, since only Atg17-Atg29-Atg31 was suggested to dissociate upon Atg1-mediated phosphorylation (Rao et al., 2016). We thus systematically examined the effect of Atg1-mediated phosphorylation on the integrity of Atg1 assemblies required for bulk and selective autophagy (Atg1-Atg13-Atg17-Atg29-Atg31 and Atg1-Atg13, respectively). Specifically, we incubated Atg1 complexes containing either WT Atg1 or catalytically inactive Atg1^D211A^ in the presence of ATP/Mg^2+^ and selectively pulled on different subunits. Affinity-purifying Atg1 after incubating Atg1-based complexes with ATP/Mg^2+^ mainly retrieved hyperphosphorylated Atg1, losing both Atg13 and Atg17-Atg29-Atg31 (Figures 3A and S3B). Likewise, pulling on Atg13 mainly retrieved Atg13, while Atg1 and Atg17-Atg29-Atg31 were largely lost (Figure S3C). Consistently, both Atg1 and Atg13 were absent when purifying the Atg17-Atg29-Atg31 complex (Figures 3B and S3D). These data suggest that Atg1-mediated phosphorylation not only ejects Atg17-Atg29-Atg31 but also disrupts the Atg1-Atg13 interaction, leaving Atg1, Atg13, and Atg17-Atg29-Atg31 as dissociation products. Importantly, treating disassembled Atg1 complex preparations with PP2A^Rts1^ or λ-PP promoted Atg1 complex reassembly (Figure 3C), implying that Atg1-mediated complex disassembly is reversible.

To understand whether Atg1 needs to be part of the complex to trigger disassembly, we added sub-stoichiometric amounts of WT Atg1 to a preformed, catalytically inactive Atg1^D211A^ complex. Strikingly, the addition of WT Atg1 dissociated an otherwise stable complex, demonstrating that phosphorylation in *trans* is sufficient to trigger complex disassembly (Figure S3E). Notably, Tpk1 was unable to destabilize inactive Atg1 complexes (Figures S3F and S3G), although it phosphorylated Atg13 *in vitro* (Figure S1J) and *in vivo* (Stephan et al., 2009).

To test whether Atg1 kinase activity also affects Atg1 complex dynamics *in vivo*, we carried out fluorescence recovery after photobleaching (FRAP) experiments. Atg13-neonGreen fluorescence rapidly recovered at the PAS in *atg11Δ* cells expressing WT Atg1, but not in cells expressing Atg1^D211A^ (Figure 3D). Moreover, Atg13 and all other Atg1 complex subunits strongly accumulated at the PAS in starved *atg1*^*D211A*^ cells (Figures 3E and 3F). This accumulation was not caused by a general block in autophagy, as deletion of Atg8 or Atg9 had no effect (Figure 3E). Notably, Atg13 PAS accumulation was also detected in *atg1*^*D211A*^ cells grown in nutrient-rich medium (Figure 3E), suggesting that kinase activity may also regulate Atg1 complex dynamics during selective autophagy.

To investigate the underlying mechanism of Atg1 complex disassembly, we studied how the phosphorylation of all three dissociation products affects their subunit-subunit interactions. While phosphorylation of Atg1 and Atg17-Atg29-Atg31 did not interefere with Atg13 binding, phosphorylation of Atg13 abolished both Atg1 and Atg17-Atg29-Atg31 binding (Figures 3G and S3D), suggesting that Atg1-mediated phosphorylation of Atg13 drives Atg1 complex disassembly.

Atg13 is also phosphorylated by TORC1 under nutrient-rich conditions inhibiting Atg17-Atg29-Atg31 complex binding and thus bulk autophagy (Fujioka et al., 2014; Kamada et al., 2010; Yamamoto et al., 2016). To examine whether Atg1 uses the reported TORC1 phosphorylation sites to eject Atg17-Atg29-Atg31, we assembled an Atg1 complex containing an Atg13 mutant with the TORC1 phosphorylation sites known to regulate Atg17 binding (S379, S428, and S429) mutated to alanine (Atg13^TOR_S^^>^^A^) (Fujioka et al., 2014; Kamada et al., 2010; Yamamoto et al., 2016; Chew et al., 2015). The resultant Atg1 complex had no obvious disassembly defect in the presence of ATP/Mg^2+^ (Figure S3H), implying that Atg1 uses a mechanism distinct from TORC1 to eject Atg17-Atg29-Atg31 during bulk autophagy.

Atg13 contains a HORMA domain in its N terminus that may act as a phosphorylation sensor (Jao et al., 2013). However, an Atg1 complex lacking the HORMA domain had no obvious disassembly defects (Figure S3I). Likewise, an Atg1 complex containing an Atg13 truncation mutant lacking both the N-terminal HORMA domain and the C-terminal region (Atg13^ΔHORMA_ΔC^) could still dissociate upon Atg1-mediated phosphorylation regardless of whether we mutated the three TORC1 phosphorylation sites in the Atg17-Atg13 interface (Atg13^ΔHORMA_ΔC_TOR_S^^>^^A^) (Figures 3H and S3J–S3L). Importantly, mass spectrometry analysis of Atg13 detected multiple phosphorylation sites in this central region that are phosphorylated by Atg1 *in vitro* (Figure 3H; Table S1), with most of them also phosphorylated *in vivo* (Figure 3H) (Fujioka et al., 2014; Hu et al., 2019; Lanz et al., 2021). Mutating the main Atg1-dependent phosphorylation sites outside the Atg1 binding region (Atg13^ΔHORMA_ΔC_pS>A^) (Figure S3J) was not sufficient to stabilize the Atg1 complex (Figure S3L). As this Atg13 mutant still underwent an electrophoretic mobility shift upon Atg1-mediated phosphorylation (Figure S3L), we mutated all of the serines and threonines within this region to alanine (Atg13^ΔHORMA_ΔC_S/T^^>^^A^), stabilizing the resultant complex in the presence of ATP/Mg^2+^ (Figure S3M). Next, we assembled an Atg1 complex with the same set of mutations in full-length Atg13 (Atg13^S/T^^>^^A^). While the resulting complex was still able to eject Atg17-Atg29-Atg31, Atg1 remained bound to Atg13^S/T>A^ even in the presence of ATP/Mg^2+^ (Figures 3I, S4A, and S4B), establishing that phosphorylation of the Atg13 central region by Atg1 specifically regulates Atg1-Atg13 dissociation. Notably, we did not detect significant changes in substrate phosphorylation between Atg1, Atg1-Atg13, and Atg1-Atg13^S/T^^>^^A^ (Figure S4C), demonstrating that Atg13^S/T>A^ binding does not change Atg1 kinase activity.

Consistent with its ability to support Atg1 complex assembly (Figures 3I, S4A, and S4B), Atg13^S/T^^>^^A^ formed a dot-like structure adjacent to the vacuole similar to WT Atg13 (Figure S4D). Likewise, both Atg13^WT^ and Atg13^S/T^^>^^A^ accumulated at the PAS in cells expressing catalytically inactive Atg1^D211A^ (Figure S4D). Atg13^S/T^^>^^A^, however, did not accumulate at the PAS in WT cells (Figure S4D), as Atg13 PAS recruitment depends on Atg17 (Suzuki et al., 2007; Cheong et al., 2008) (Figure S4E) and Atg13^S/T^^>^^A^ dissociation from Atg17 was largely unimpaired (Figures 3I and S4A). As a consequence, neither Atg17 nor Atg1 accumulated at the PAS upon starvation in cells expressing Atg13^S/T>A^ (Figure S4F). These data suggest that Atg13^S/T>A^ promotes assembly of an active Atg1 complex at the PAS, which is specifically impaired in the phosphorylation-mediated dissociation of the Atg1-Atg13^S/T>A^ subcomplex.

To examine the physiological importance of Atg1-Atg13 complex dissociation, we studied bulk and selective autophagy in Atg13^S/T>A^-expressing cells. Strikingly, bulk autophagy was strongly impaired in *atg13*^*S/T*^^*>*^^*A*^ cells, while the selective cytoplasm-to-vacuole targeting (Cvt) pathway was largely unaltered (Figures 3J–3L and S4G).

These results demonstrate that the Atg1 complex is a highly dynamic entity that needs to cycle between an assembled and a disassembled state, driven at least in part by Atg13 phosphorylation and counteracting dephosphorylation (Figure 3M).

### Atg1 kinase activity inhibits Atg8 lipidation

**Figure 4:**
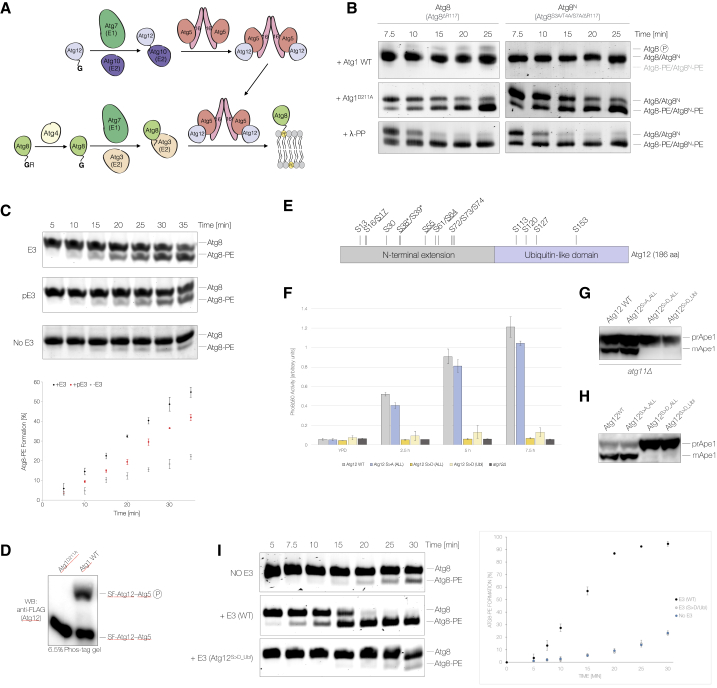
Atg8 lipidation is inhibited by Atg1-mediated phosphorylation of the E3 (A) Schematic overview of Atg8 processing and lipidation. (B) Recombinant Atg3, Atg7, and Atg12–Atg5-Atg16 were incubated with WT Atg1, Atg1^D211A^, or λ-PP in the presence of ATP/Mg^2+^ and PP inhibitors. Liposomes were added and lipidation reactions were started by the addition of Atg8 (Atg8^ΔR117^) or Atg8^N^ (Atg8^S3A/T4A/S7A/ΔR117^). Samples were taken at indicated time points and analyzed by urea-SDS-PAGE and Sypro Ruby staining. (C) Atg8 lipidation was monitored in the presence or absence of E3 and in the presence of an E3 pre-phosphorylated by Atg1 (pE3). Lipidation reactions were set up as illustrated in Figure S5G. Samples were taken and analyzed as in (B). The average lipidation ± standard deviation is plotted for each time point (n = 3; bottom panel). (D) Endogenous SF-tagged Atg12 was purified from nitrogen-starved yeast expressing WT Atg1 or Atg1^D211A^. The electrophoretic mobility of the Atg12–Atg5 conjugate was compared by Phos-tag SDS-PAGE and WB. (E) Schematic overview of *S. cerevisiae* Atg12 highlighting the N-terminal extension, Ubl domain, and Atg1-dependent *in vitro* phosphorylation sites. Asterisks indicate phosphorylation sites also identified *in vivo* (Hu et al., 2019; Lanz et al., 2021). Underlined sites were previously reported to be phosphorylated by Atg1 *in vitro* (Hu et al., 2019). (F) Bulk autophagy was quantified in Atg12-, Atg12^S>A_ALL^-, Atg12^S>D_ALL^-, and Atg12^S>D_Ubl^-expressing or *atg12Δ* cells using the Pho8Δ60 assay. Cells were exponentially grown in YPD medium or nitrogen starved for 2.5, 5, or 7.5 h. (G) Bulk autophagy was monitored in nitrogen-starved *atg11Δ* cells expressing WT Atg12, Atg12^S^^>^^A_ALL^, Atg12^S^^>^^D_ALL^, or Atg12^S^^>^^D_Ubl^. Ape1 processing was monitored by WB. (H) The Cvt pathway was analyzed in cells expressing Atg12^WT^, Atg12^S^^>^^A_ALL^, Atg12^S^^>^^D_ALL^, or Atg12^S^^>^^D_Ubl^ by monitoring Ape1 processing. (I) Atg8 lipidation was monitored in the presence of an E3 containing either WT Atg12 or Atg12^S^^>^^D_Ubl^. Atg8 lipidation was analyzed as in (B).

Our *in vitro* kinase assays showed that several components of the two interconnected Ubl protein-conjugating systems are phosphorylated by Atg1 (Figures 1C, 4A, S1F, S1K, and S1L), suggesting that Atg1 may regulate Atg8 lipidation. Atg8 lipidation was blocked when we prephosphorylated all of the enzymes involved in Atg8 lipidation (Atg7, Atg3, and Atg12–Atg5-Atg16) using Atg1 (Figures 4B and S5C). To distinguish a bona fide inhibition from an electrophoretic mobility shift caused by Atg1-mediated Atg8-PE phosphorylation (Figure S5D), we generated an N-terminal alanine mutant, Atg8^S3A/T4A/S7A/ΔR117^ (Atg8^N^), which is fully functional *in vitro* (Figure S5E) and *in vivo* (Figure S5F), but can no longer undergo a phosphorylation-dependent upshift upon lipidation (Figure S5D). Atg8^N^ lipidation was still blocked in the presence of Atg1 kinase activity, confirming the inhibition of Atg8 lipidation by Atg1 (Figures 4B and S5C).

### Atg1-dependent phosphorylation of Atg12 inhibits E3 activity

To dissect the mechanism of this inhibition, we individually phosphorylated the different Atg1 targets. We purified Atg1 phosphorylated Atg12–Atg5-Atg16, allowing us to carry out Atg8 lipidation assays in the absence of Atg1. Atg8 lipidation was delayed when phosphorylated but not dephosphorylated E3 was added (Figures 4C and S5G), suggesting that Atg1 directly inhibits the E3. The Atg5-Atg16 complex is not active as E3 unless conjugated to Atg12 (Hanada et al., 2007; Metlagel et al., 2013). However, neither E3 formation nor stability were affected by Atg1 kinase activity (Figures S5A and S5B). As Atg12 and Atg12–Atg5 are both Atg1 targets (Figure 1C), we investigated the role of Atg1-dependent Atg12 phosphorylation. We purified endogenous Atg12 from nitrogen-starved yeast expressing either WT Atg1 or catalytically inactive Atg1^D211A^ and compared the electrophoretic mobility of the Atg12–Atg5 conjugate by Phos-tag SDS-PAGE. Only the Atg12–Atg5 conjugate purified from WT cells migrated as two distinct bands, with a unique slower migrating band consistent with Atg1-dependent Atg12 phosphorylation *in vivo* (Figure 4D). Mass spectrometry analysis of *in vitro* phosphorylated Atg12 identified 12 Atg1 phosphorylation sites in the N-terminal extension and 4 in the Ubl domain (Figure 4E; Table S1). Consistently, mutation of these phosphorylation sites strongly reduced the Atg1-mediated phosphorylation of Atg12 (Figure S5H), confirming that we identified the majority of the Atg1-dependent phosphorylation sites.

To test their functional significance, we compared bulk and selective autophagy in cells expressing phosphorylation-deficient and phosphomimicking mutants, Atg12^S^^>^^A_ALL^ and Atg12^S^^>^^D_ALL^, respectively. While Atg12^S^^>^^A_ALL^-expressing cells showed only a mild bulk autophagy defect (Figures 4F and 4G) and a fully functional Cvt pathway (Figure 4H), both autophagy pathways were completely blocked in the presence of Atg12^S^^>^^D_ALL^ (Figures 4F–4H), likely resulting from an Atg8 lipidation defect *in vivo* (Figure S5I). Mutating the four phosphorylation sites in the Ubl domain to aspartate (Atg12^S^^>^^D_Ubl^) fully recapitulated the observed defects in Atg8 lipidation, bulk, and selective autophagy (Figures 4F–4H and S5I), while a phosphomimicking mutant of the N-terminal phosphorylation sites had no significant defect (Figures S5J–S5L).

To corroborate these findings, we assembled an E3 complex containing the Atg12^S^^>^^D_Ubl^ mutant *in vitro*. Although Atg12^S^^>^^D_Ubl^ was efficiently conjugated to Atg5, allowing the formation of a phosphomimicking E3 (Figure S5M), the resultant E3 failed to stimulate Atg8 lipidation (Figure 4I). We therefore conclude that the phosphorylation of the Ubl domain of Atg12 by Atg1 efficiently blocks Atg8 lipidation, explaining at least in part the strong autophagy and Atg8 lipidation defect observed in Atg12^S^^>^^D_Ubl^-expressing cells.

### Atg1-mediated phosphorylation of Atg3 inhibits Atg8 lipidation

**Figure 5:**
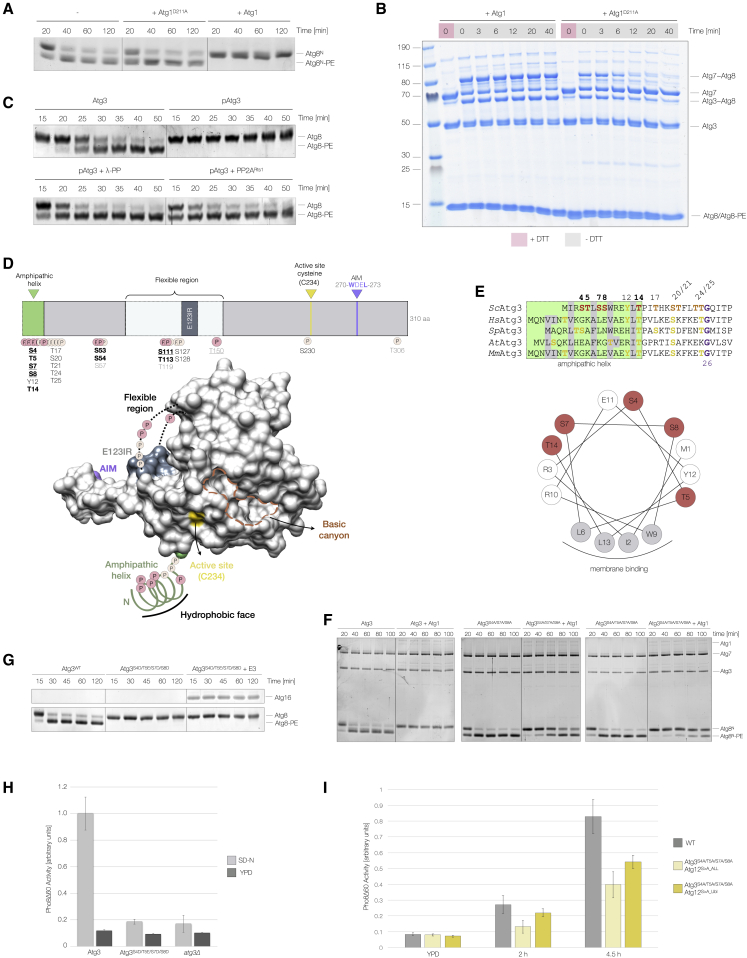
Atg8 lipidation is inhibited by Atg1-mediated phosphorylation of Atg3 (A) Atg3 and Atg7 were incubated with either WT Atg1 or Atg1^D211A^ in the presence of ATP/Mg^2+^. Liposomes were added and lipidation reactions were started by the addition of Atg8^N^. Lipidation was analyzed by urea-SDS-PAGE and Coomassie staining. (B) Atg3, Atg7, and Atg8 were individually incubated with WT Atg1 or Atg1^D211A^ and ATP/Mg^2+^ before combining to promote Atg3 charging. Liposomes were added to monitor Atg8 discharge in a time-dependent manner. Samples were analyzed by SDS-PAGE under reducing or non-reducing conditions. (C) Atg8 lipidation assays were carried out using WT Atg3, Atg1-phosphorylated Atg3 (pAtg3), or λ-PP or PP2A^Rts1^-treated pAtg3. Samples were taken at the indicated time points, and Atg8 lipidation was analyzed by urea-SDS-PAGE and Sypro Ruby staining. (D) Schematic domain overview and surface representation of *S. cerevisiae* Atg3 (PDB: 2DYT) highlighting phosphorylation sites and unique functional elements, including the N-terminal amphipathic helix (green), the E1, E2, and E3 interacting region (E123IR), the active site cysteine, and the AIM. Sites phosphorylated *in vivo* and by Atg1 *in vitro* are highlighted in bold black. *In vivo* phosphorylation sites regulated by Atg1 are underlined. Phosphorylation sites detected either *in vitro* or *in vivo* are shown in gray and black, respectively. (E) Sequence alignment of the Atg3 N terminus with Atg1-dependent *in vitro* and *in vivo* phosphorylation sites colored in red and orange. Residues in yellow indicate potential phosphorylation sites in other organisms. The helical wheel representation of the N-terminal amphipathic helix is shown below. (F) Atg8^N^ lipidation was compared in the presence or absence of Atg1 using either WT Atg3, Atg3^S4A/S7A/S8A^, or Atg3^S4A/T5A/S7A/S8A^. Atg7, Atg3, and Atg8^N^ were separately incubated with or without Atg1 before starting the lipidation reaction. Samples were analyzed by urea-SDS-PAGE and Sypro Ruby staining. (G) Atg8 lipidation was studied in the presence of Atg3 or Atg3^S4D/T5E/S7D/S8D^. In a separate reaction, the E3 was added to Atg3^S4D/T5E/S7D/S8D^-containing reactions. Atg8 lipidation was analyzed as in (F). (H) Bulk autophagy was quantified in nitrogen-starved (4 h) WT Atg3, *atg3Δ*, and Atg3^S4D/T5E/S7D/S8D^-expressing cells using the Pho8Δ60 assay (n = 3). (I) The Pho8Δ60 assay was used to quantify bulk autophagy in yeast co-expressing Atg3^S4A/T5A/S7A/S8A^ with either Atg12^S^^>^^A_ALL^ or Atg12^S^^>^^A_Ubl^ (n = 3).

To examine whether E3 phosphorylation is the sole reason for the observed inhibition of Atg8 lipidation, we took advantage of the fact that Atg8 lipidation does not require E3 activity *in vitro*. Strikingly, Atg1-mediated phosphorylation efficiently blocked Atg8 lipidation even in the absence of the E3 (Figure 5A). This inhibition was specific to Atg1, as addition of other kinases (PKA, Tpk1, Hrr25, and Plk1) did not alter Atg8 lipidation (Figure S6A). Atg1 kinase activity did not affect the formation of the thioester-linked Atg3-Atg8 intermediate (Figure S6B), demonstrating that Atg1 neither regulates E1 activity nor transthiolation nor Atg3 charging. We therefore tested Atg8 discharge to PE-containing liposomes and observed a striking discharge defect in the presence of Atg1 kinase activity (Figure 5B).

To confirm that Atg3 phosphorylation specifically inhibits Atg8 lipidation, we pre-phosphorylated Atg3 using Atg1 (pAtg3) and further purified it to avoid simultaneous Atg8 and Atg7 phosphorylation in Atg8 lipidation assays. Strikingly, pAtg3 was not able to lipidate Atg8, and this effect could be reversed by incubating pAtg3 with either λ-PP or PP2A^Rts1^ (Figure 5C). To further investigate the molecular mechanism of Atg3 inhibition, we mapped the Atg1-dependent phosphorylation sites *in vitro* and *in vivo*. Mass spectrometry analysis showed that the majority of Atg1-dependent phosphorylation sites localize to the E1, E2, and E3 interacting region and unique N-terminal extension (Figures 5D, 5E, S6C–S6G; Table S1). Only when we mutated the N-terminal phosphorylation sites, but not those in the E1, E2, and E3 interacting region, to alanine (Atg3^S4A/S7A/S8A^ and Atg3^S4A/T5A/S7A/S8A^) could we partially rescue Atg1-mediated inhibition of Atg8 lipidation (Figures 5F, S6H, and S6I).

Consistent with these results, the corresponding phosphomimicking mutant (Atg3^S4D/T5E/S7D/S8D^) completely abolished Atg8 lipidation *in vitro*, and this effect could not be rescued by the addition of E3 (Figure 5G). Consistently, cells expressing the Atg3^S4D/T5E/S7D/S8D^ mutant were unable to promote bulk autophagy (Figure 5H) and the selective Cvt pathway (Figure S6J), and they were unable to lipidate Atg8 *in vivo* (Figure S6K). We also analyzed bulk autophagy in cells in which Atg1-mediated inhibition of Atg8 lipidation is impaired. Despite the residual inhibition of the Atg3 alanine mutants and coexisting E3 inhibition, bulk autophagy was slightly reduced in Atg3^S4A/S7A/S8A^- and Atg3^S4A/T5A/S7A/S8A^-expressing cells (Figure S6L). Importantly, this defect was exacerbated by co-expressing Atg3^S4A/T5A/S7A/S8A^ and the phosphorylation-deficient Atg12^S^^>^^A_ALL^ or Atg12^S^^>^^A_Ubl^ mutants (Figure 5I), suggesting that Atg1-dependent downregulation of Atg8 lipidation is important for bulk autophagy *in vivo*.

The N terminus of Atg3 comprises an amphipathic alpha helix (Figures 5D and 5E), which mediates membrane binding (Hanada et al., 2009; Hervás et al., 2017; Nath et al., 2014). The N-terminal Atg1 phosphorylation sites map largely to the hydrophilic face of the amphipathic helix (Figure 5E), making them unlikely to directly interfere with membrane binding. Atg1-dependent phosphorylation did not significantly change the binding of Atg3 to Atg8-PE-containing liposomes (Figure S6M). Notably, the N terminus of Atg3 is positioned in close proximity to the active site (Figures 5D and S6N). Adjacent to the active site is a highly conserved, positively charged canyon that coordinates two sulfate ions in the Atg3 crystal structure (Yamada et al., 2007) (Figures 5D and S6N–S6Q), which can suggest the binding of phosphoryl groups. A phosphorylated N terminus could provide a high density of negatively charged phosphoryl groups to engage with the positively charged canyon, thereby blocking the active site. Consistent with such a model, the presence of negatively charged peptides is sufficient to inhibit Atg8 lipidation *in vitro* (Figure S6R). Glycine 26, which is invariant among Atg3 orthologs (Figures 5E and S6N), could act as a hinge to provide the N terminus with enough flexibility to mediate binding to the basic canyon. Rigidifying this pivot by replacing the highly conserved glycine with a proline completely abolished Atg3 activity (Figure S6H). These data suggest that Atg1-mediated phosphorylation of the Atg3 N terminus induces a conformational change that blocks the E2 active site, and as a result, inhibits Atg8 discharge.

## Discussion

To better understand the complex role of Atg1 in orchestrating autophagy, we combined *in vitro* reconstitution with cell-based assays to systematically study the functional consequences of Atg1-mediated phosphorylation. We find that Atg1 autophosphorylation and substrate phosphorylation are both highly upregulated by Atg8-PE. Atg1 autophosphorylation stimulates membrane recruitment of Atg1 and promotes the phosphorylation of phagophore-associated Atg1 substrates. Surprisingly, Atg1 inhibits Atg8 lipidation by phosphorylating the Atg8-specific E2 and E3, thereby limiting the Atg8-PE density in the growing phagophore. Atg1 recruitment by Atg8-PE therefore establishes both positive and negative feedback. Finally, Atg1-mediated phosphorylation of Atg13 triggers rapid disassembly of Atg1-based complexes at the PAS, resulting in the dissociation of Atg17-Atg29-Atg31 and Atg1 from Atg13, with the latter playing an important role during bulk autophagy. Since Atg1 complex disassembly and inhibition of Atg8 lipidation can be reversed by PP activity, we conclude that autophagy is driven by the spatiotemporal regulation of phosphorylation and counteracting dephosphorylation events.

Atg1 complex formation appears to be spatiotemporally regulated as Atg13 and Atg17 only localize to the PAS and are absent from growing autophagosomal membranes, despite Atg8-PE-dependent recruitment of Atg1 (Suzuki et al., 2013). We show that Atg1 complexes at the PAS are intrinsically unstable due to Atg1-mediated phosphorylation of Atg13, which triggers its dissociation from Atg1 and Atg17-Atg29-Atg31. Despite the overall desphosphorylation of Atg13 in response to nutrient starvation (Kamada et al., 2000), our data suggest that Atg1 continuously phosphorylates Atg13 and thus destabilizes the Atg1 complex even in the absence of TORC1-mediated phosphorylation. PAS localized PPs dephosphorylating Atg13 (Memisoglu et al., 2019) are thus likely to drive the rapid reassembly of Atg1-based complexes. It is tempting to speculate that the resultant Atg1 complex dynamics not only provide the molecular basis for the liquid-like properties of the PAS (Fujioka et al., 2020) but also drive autophagosomal membrane formation. While Atg1 complex assembly is thought to tether Atg9-containing vesicles (Rao et al., 2016), dissociation of the Atg1 complex may promote the release of membrane fusion intermediates, freeing up Atg9 binding sites to drive rapid phagophore expansion aided by Atg2-dependent lipid transfer (Matoba et al., 2020; Osawa et al., 2019; Valverde et al., 2019; Maeda et al., 2019). The ejection of Atg17-Atg29-Atg31 may also shape autophagosomal membranes (Bahrami et al., 2017) or help terminate autophagy by continuously exposing the TORC1 phosphorylation sites in Atg13 (Fujioka et al., 2014; Yamamoto et al., 2016), thereby allowing rapid sensing of TORC1 reactivation upon nutrient replenishment.

Mutational analysis revealed the functional significance of Atg1 complex dissociation, as cells expressing an Atg13 mutant unable to dissociate from Atg1 are largely defective for bulk autophagy. As these cells are proficient in forming smaller Cvt vesicles under nutrient-rich conditions, our work highlights further mechanistic differences between bulk and selective autophagy. Given the strong conservation of Atg1 and Atg13, the described oscillatory behavior of human Atg13 during mitophagy (Dalle Pezze et al., 2021), and the accumulation of Ulk1 in the presence of Ulk1 inhibitors (Petherick et al., 2015), it is likely that similar mechanisms also regulate Atg1 complex dynamics in higher eukaryotes.

In addition to Atg1 complex dynamics and autoactivation (Kijanska et al., 2010; Yeh et al., 2010) Atg1 phosphorylation also stimulates its own recruitment to growing autophagosomal membranes by exposing at least one additional phosphorylation-regulated AIM. Atg8-PE binding further upregulates Atg1 autophosphorylation and substrate phosphorylation. The resultant high-affinity/high-activity state leads to the phosphorylation, recruitment, and activation of other Atg1 molecules, creating a zone of high Atg1 kinase activity along the growing phagophore. A concomitant decrease in PP activity away from the PAS, as judged by the inability of phagophore-bound Atg1 to (re)associate with Atg13 and Atg17 (Suzuki et al., 2013), may further enhance this positive feedback. While Atg1 activation at the PAS requires Atg13- and Atg17-dependent clustering of Atg1 complexes (Yamamoto et al., 2016), Atg8-PE-induced activation of Atg1 provides a means to sustain high Atg1 kinase activity along growing phagophores in the absence of Atg13 and Atg17. Most Atg1 substrates are membrane associated, containing either AIMs or other membrane-targeting regions (Baskaran et al., 2012; Birgisdottir et al., 2019; Bozic et al., 2019; Herman et al., 1991; Kaufmann et al., 2014; Ngu et al., 2015; Romanov et al., 2012; Rostislavleva et al., 2015; Suzuki et al., 2013). Hence, Atg8-PE-containing membranes act as scaffolds to recruit both Atg1 and its substrates. This increases their local concentrations and restrains diffusion to a two-dimensional surface, thereby increasing substrate phosphorylation along the growing autophagosomal membrane. This mechanism is distinct from the initial stages of Atg1 activation during selective autophagy, when the cargo itself clusters and activates Atg1 with the help of cargo adaptors and the selectivity factor Atg11 (Kamber et al., 2015). Nevertheless, it is conceivable that Atg8-PE-dependent Atg1 activation also contributes to Atg1 activation during selective autophagy, but akin to bulk autophagy, this activation mechanism would act downstream of Atg8 lipidation.

Atg8-PE not only recruits Atg1 but also the Atg8-specific E2 and E3 to growing phagophores (Ngu et al., 2015; Suzuki et al., 2013). Surprisingly, we find that Atg1-mediated phosphorylation downregulates their enzymatic activities. This Atg1-mediated negative feedback is functionally important *in vivo*, since bulk autophagy is strongly impaired in cells co-expressing phosphorylation-deficient Atg3 and Atg12 mutants. High levels of Atg8-PE have been shown to affect membrane morphology, resulting in local membrane deformations (Knorr et al., 2014). Hence, self-limiting Atg8-PE levels in the forming autophagosomal membrane by phosphorylating two of the key enzymes may provide a means to control phagophore expansion, preventing membrane deformations. Eukaryotic cells also need to be resourceful with their available Atg8 pool, especially when facing long periods of starvation. Autophagosome formation severely reduces the pool of free Atg8 since a substantial portion of Atg8-PE is degraded in the vacuole together with the inner autophagosomal membrane. Self-limiting Atg8 lipidation may thus reflect a need for keeping a sufficiently large Atg8 pool. Consistently, Atg8 protein levels are upregulated upon bulk autophagy induction (Xie et al., 2008), and mislocalized Atg8-PE is continuously retrieved from non-autophagosomal membranes by an Atg4-dependent proofreading mechanism replenishing soluble Atg8 (Nakatogawa et al., 2012a).

Since Atg8 lipidation is essential (Tsukada and Ohsumi, 1993; Ichimura et al., 2000), mechanisms must exist to spatiotemporally counteract Atg1-dependent inhibition. As Atg3 and Atg12–Atg5-Atg16 inhibition is reversible, it is likely that PPs located at the PAS (Memisoglu et al., 2019; Wong et al., 2015; Yeasmin et al., 2016) promote Atg8 lipidation by dephosphorylating both E2 and E3, allowing accumulation of Atg8-PE in the growing phagophore. This recruits Atg1, which in turn downregulates Atg8-PE formation by inhibiting the E2 and E3. Such negative feedback allows for the self-regulation of Atg8-PE levels in the growing autophagosomal membrane, where Atg8-PE is further stabilized by Atg1-mediated inhibition of Atg4 (Pengo et al., 2017; Sánchez-Wandelmer et al., 2017).

While E3 inhibition is at least in part mediated by Atg1-mediated phosphorylation of the Atg12 Ubl domain, Atg3 inhibition is mediated by phosphorylation of its N-terminal extension, preventing Atg8 discharge to PE. Our data suggest a model in which phosphorylation of the flexible N terminus blocks the Atg3 active site by promoting an electrostatic interaction with the adjacent highly conserved basic canyon. Atg1-mediated inhibition of Atg3 is distinct from the recently described Atg3 auto-inhibition, which uses the E1, E2, and E3 interacting region to restrain the conformation of the catalytic loop in Atg3 in a phosphorylation-independent manner (Zheng et al., 2019). This E1, E2, and E3 interacting region-dependent auto-inhibition protects from spurious discharge to non-autophagosomal membranes since it couples Atg8 lipidation to E3 binding. In contrast, Atg1-mediated inhibition of Atg3 cannot be reversed by the E3, requiring instead PP activity. Atg1 can thus inhibit Atg8 lipidation, even in the presence of the E3, and therefore regulate Atg8 lipidation along the growing phagophore where Atg3 and the E3 co-localize (Ngu et al., 2015; Suzuki et al., 2013).

Our work emphasizes how *in vitro* reconstitution approaches can help us understand complex intracellular signaling networks at the molecular level. It paves the way for more detailed mechanistic studies investigating the spatiotemporal regulation of bulk and selective autophagy by both protein kinase and phosphatase signaling.

### Limitations of the study

While our results clearly show that the Atg1 complex is a highly dynamic entity, mediated at least in part by Atg1-dependent Atg13 phosphorylation and counteracting dephosphorylation, further work is required to understand why its continuous dissociation and reassembly is required for bulk autophagy but dispensible for selective autophagy.

Moreover, although we are starting to understand the physiological importance of self-regulating Atg8-PE levels, more advanced microscopy experiments are needed to visualize the morphological consequences of unbalanced Atg8 lipidation in cells.

Finally, although co-expressing the phosphorylation-deficient Atg3^S4A/T5A/S7A/S8A^ and Atg12^S^^>^^A_ALL^ mutants results in a strong bulk autophagy reduction, it is likely that fully disabling Atg1-mediated E2 and E3 inhibition will exhibit an even more pronounced autophagy defect, aiding future studies. Identifying an Atg1 mutant that can no longer interact with Atg8-PE regardless of its phosphorylation status may be an alternative means to study autophagy in cells unable to self-limit Atg8 lipidation and to trigger positive feedback. However, we still lack a catalytically active Atg1 mutant that has fully lost its ability to bind Atg8-PE, regardless of its phosphorylation status. The previously characterized Atg1 AIM mutant is likely to underestimate the physiological relevance of recruiting and activating Atg1 along the growing phagophore, restricting its use.

## STAR★Methods

### Key resources table

**Table:** 

REAGENT or RESOURCE	SOURCE	IDENTIFIER
**Antibodies**		

Goat anti-Mouse IgG (H + L)-HRP	Biorad	Cat#1706516
Goat anti-Rabbit IgG (H + L)-HRP	Biorad	Cat#1706515
Mouse monoclonal anti-FLAG M2 antibody	Sigma	F1804
Rabbit polyclonal anti-Atg8 antibody	Papinski et al., 2014	N/A
Rabbit polyclonal anti-Ape1 antibody	Kraft et al., 2012	N/A

**Bacterial and virus strains**		

Subcloning Efficiency DH5α Compentent Cells	Thermo Fisher	Cat#18265-017
Rosetta 2 Competent Cells	Novagen	Cat#71402
DH10Multibac Cells	Schreiber et al., 2011	N/A
BL21-CodonPlus (DE3)-RIL Competent Cells	Agilent Technologies	Cat#230245

**Chemicals, peptides, and recombinant proteins**		

NaCl	Sigma	Cat#S9888
1,4-Dithiothreitol (DTT)	Melford	Cat#D11000
HEPES	Sigma	Cat#H3375
MES	Sigma	Cat#M8250
UREA	Invitrogen	Cat#AM9902
EDTA	Sigma	Cat#E5134
Ammonium bicarbonate	Sigma	Cat#A6141
Glucose	Sigma	Cat#7021
Glycerol	Sigma	Cat#G5516
Acetic acid	Sigma	Cat#695092
Chloroform	Alfa Aesar	Cat#11398187
Acetone	Sigma	Cat#179124
Acetonitrile	Thermo Fisher Scientific	Cat#51101
Iodoacetamide	Thermo Fisher Scientific	Cat#39271
Diethyl ether	Sigma	Cat#179272
Isopropanol	Sigma	Cat#I9516
G418 solution	Sigma	Cat#G8168
clonNAT	Jena Bioscience	AB-102XL
Zeocin	VWR	Cat#67140
Ampicillin	Sigma	Cat#A9393
Gentamicin sulfate	Sigma	Cat#G1914
Kanamycin sulfate	Thermo Fisher Scientific	Cat#11815032
Tetracycline	Alfa Aesar	Cat#J61714
Chloramphenicol	Sigma	Cat#C0378
Bluo-gal	Invitrogen	Cat#15519028
IPTG	Invitrogen	Cat#15529019
Penicillin-Streptomycin-Glutamine	GIBCO	Cat#10378016
G418 solution	Sigma	Cat#G8168
clonNAT	Jena Bioscience	AB-102XL
Zeocin	VWR	Cat#67140
DMSO	Sigma	Cat#D8418
Trichloroacetic acid	Sigma	Cat#T6399
Formic acid	Fisher Scientific	Cat#10596814
TCEP	Thermo Fisher Scientific	Cat#77720
DDM	Anatrace	Cat#D310HA
LDAO	Avanti	Cat#850545P
BODIPY TMR C_5_-Maleimide	Thermo	Cat#B30466
ATP	Sigma	Cat#A2383
[γ-^32^P]-ATP	Hartmann Analytics	SRP301
*d*-Desthiobiotin	Sigma	Cat#D1411
dNTPs	Thermo Fisher Scientific	Cat#R0193
L-Glutathione reduced	Sigma	Cat#G4251
p-Nitrophenyl Phosphate	NEB	P0757
L-a-Phosphatidylethanolamine	Avanti	Cat#840026
L-a-Phosphatidylinositol	Avanti	Cat#840042
L-a-Phosphatidylcholine	Avanti	Cat#840055
L-a-Phosphatidylserine	Avanti	Cat#840032
PageRuler Plus Prestained Protein ladder	Thermo Fisher Scientific	Cat#26620
cOmplete EDTA-free Protease Inhibitor Cocktail	Roche	5056489001
PhosSTOP phosphatase inhibitors	Roche	4906837001
Yeast nitrogen base without amino acids and ammonium sulfate	Millipore	Cat#Y1251
PMSF	Thermo Fisher Scientific	Cat#36978
Leupeptin	Thermo Fisher Scientific	Cat#78435
Pepstatin A	Thermo Fisher Scientific	Cat#78436
Benzamidine hydrochloride hydrate	Sigma	Cat#6506
c-Myc-peptide	Thermo Fisher Scientific	Cat#20170
3xFLAG peptide	Generon	Cat#A6001
GeneJuice	Sigma	Cat#70967
Lambda protein phosphatase	NEB	Cat#P0753
Apyrase	NEB	Cat#M0398
Pierce Universal nuclease	Thermo Fisher Scientific	Cat#88701
cAMP-dependent Protein Kinase (PKA) catalytic subunit	NEB	Cat#P6000
Polo-like kinase 1 (Plk1)	SignalChem	Cat#P41-10H
Trypsin	Thermo Fisher Scientific	Cat#90058
Ubiquitin	R&D Systems	Cat#U-100H-10M
Lysyl-endopeptidase (LysC)	Wako	Cat#125-05061
Phusion High-Fidelity DNA polymerase	NEB	Cat#M0530
USER enzyme	NEB	Cat#M5505
Restriction enzymes (various)	NEB	N/A

**Critical commercial assays**		

High-Select Fe-NTA Phosphopeptide Enrichment Kit	Thermo Fisher Scientific	Cat# A32992
BCA Protein Assay Kit	Thermo Fisher Scientific	Cat#23225
Zero Blunt TOPO PCR Cloning kit	Invitrogen	Cat#450245
Plasmid Miniprep Kit	Thermo Fisher Scientific	Cat#K0503
Gel Extraction Kit	Thermo Fisher Scientific	Cat#K0691

**Deposited data**		

The mass spectrometry data were deposited to the ProteomeXchange Consortium via the PRIDE partner repository with the dataset identifier PXD029047.	Perez-Riverol et al., 2019	PXD029047

**Experimental models: Cell lines**		

Sf9 insect cells	Invitrogen	Cat#10503433
High Five insect cells	Invitrogen	Cat#10747474

**Experimental models: Organisms/strains**		

All *Saccharomyces cerevisiae* strains used in this study are listed in Table S2	N/A	N/A

**Oligonucleotides**		

All DNA oligonucleotides were purchased from Sigma and are listed in Table S4	N/A	N/A

**Recombinant DNA**		

All plasmids used in this study are listed in Table S3	N/A	N/A

**Software and algorithms**		

Fiji	Schindelin et al., 2012	https://imagej.net/software/fiji/
Micromanager	Edelstein et al., 2014	https://micro-manager.org/
SeqMan Pro	DNASTAR	https://www.dnastar.com/
Chimera	UCSF	https://www.cgl.ucsf.edu/chimera/
NEBcutter V2	NEB	http://nc2.neb.com/NEBcutter2/
MaxQuant 1.6.12.0	Cox and Mann, 2008	https://www.maxquant.org/
Skyline-daily (64-bit) 20.2.1.404	MacLean et al., 2010	https://skyline.ms/
scikit-image 0.18.1	van der Walt et al., 2014	https://scikit-image.org

**Other**		

96-well glass bottom microplates	Greiner Bio-One	Cat#655891
TimsTOF Pro	Bruker Daltonics	N/A
NanoElute	Bruker Daltonics	N/A
5600 TripleTOF	Sciex	N/A
NanoLC Ultra	Sciex/Eksigent	N/A
LTQ-Orbitrap XL	Thermo Fisher Scientific	N/A
EASY-nLC	Thermo Fisher Scientific/Proxeon	N/A
Nikon Eclipse Ti2 Inverted Microscope	Nikon Instruments Inc., (2017)	N/A
Nikon Plan Apo 100X/1.45 Oil	Nikon Instruments Inc., (2017)	N/A
Photometrics Prime 95B sCMOS camera	Teledyne Photometrics	https://www.photometrics.com/
ÄKTA Pure Protein Purification System	Cytiva	N/A
EnSight Multimode Plate reader	Perkin Elmer	HH34000000
Typhoon FLA 9500	GE Healthcare	N/A
Freezer Mill 6875D	Spex SamplePrep	WZ-04577-94
Fermenter New Brunswick BioFlow 510	Eppendorf	N/A
Fermenter New Brunswick BioFlow 610	Eppendorf	N/A
C18 Sep-Pak columns	Waters	WAT023590
Superose 6 Increase 10/300 GL	Cytiva	Cat#29091596
HiLoad 16/600 Superdex 75	Cytiva	Cat#28989333
HiLoad 16/600 Superdex 200	Cytiva	Cat#28989335
Resource Q anion exchange column	Cytiva	Cat#17117901
Resource S cation exchange column	Cytiva	Cat#17118001
GST trap	Cytiva	Cat#17528201
Strep-Tactin Superflow Plus Resin	QIAGEN	Cat#30060
Strep-Tactin Superflow Plus cartridge	QIAGEN	Cat#30004
Anti-FLAG M2 Affinity gel	Sigma	Cat#A2220
Anti-c-Myc Agarose	Thermo Fisher Scientific	Cat#20168
MOPS SDS Running Buffer	Thermo Fisher Scientific	Cat#NP0001
MES SDS Running Buffer	Thermo Fisher Scientific	Cat#NP0002
Quick Coomassie Stain	Neo Biotech	Cat#NB-45-00078
Sypro Ruby Protein Gel Stain	Thermo Fisher Scientific	Cat#12000
Clarity Western ECL Substrate	Biorad	Cat#1705060
PVDF transfer membrane	Millipore	Cat#IPFL85
Nitrocellulose membrane	Thermo Scientific	Cat#88018
SF900 II Medium	GIBCO	Cat#10902104 Cat#10902104

### Resource availability

#### Lead contact

Further information and requests for reagents should be directed to and will be fulfilled by the Lead Contact Anne Schreiber (anne.schreiber@crick.ac.uk).

#### Materials availability

Plasmids generated in this study are available from the Lead Contact without restriction or require a completed Materials Transfer Agreement if there is potential for commercial application.

### Experimental model and subject details

#### *S. cerevisiae* strains and media

All yeast strains used in this study are derived from *Saccharomyces cerevisiae* BY4741 (MAT**a**; *his3Δ1*; *leu2Δ0*; *met15Δ0*; *ura3Δ0*) and are summarized in Table S2. Strains were created by transforming the linearized plasmids listed in Table S3. Yeast were grown in YPD (1% yeast extract, 2% peptone, and 2% glucose) or synthetic defined (SD) medium (0.17% yeast nitrogen base, 0.5% ammonium sulfate, 2% glucose and amino acids as required). Starvation experiments were carried out by growing yeast in nitrogen starvation (SD-N) medium (0.17% yeast nitrogen base without amino acids and ammonium sulfate and 2% glucose).

#### *E. coli* strains and media

*E. coli* (DH5α, Rosetta 2, BL21 RIL and DH10Multibac) were grown in Terrific Broth (TB) medium.

#### Insect cells and media

Insect cells (Sf9 and High Five cells; Invitrogen) were grown in Sf900 II medium (GIBCO) supplemented with 0.1X Penicillin-Streptomycin-Glutamine (GIBCO).

### Method details

#### Cloning and plasmids

All *S. cerevisiae* genes were PCR amplified from genomic DNA. Restriction sites and tags were introduced by PCR. Mutations and gene fusions were generated by splicing by overlap extension (Heckman and Pease, 2007) or USER cloning (Bitinaite et al., 2007). Gene synthesis was carried out by Eurofins Genomics. All constructs were sequence verified (GATC).

#### Plasmids used for yeast strain construction

Plasmids used for yeast strain construction are listed in Table S3. All plasmids are pCR-Blunt II or IV TOPO (Invitrogen) derived. Constructs to generate point mutations or gene fusions contained the gene specific promoter (∼500 bp upstream of the gene specific start codon) as one region of homology, the mutated open reading frame or gene fusion, the terminator sequence (∼150-300 bp downstream of the gene specific stop codon), the selection cassette and a second region of homology downstream of the terminator sequence (300-500 bp). Gene deletions were generated by fully replacing the target gene with the indicated selection cassette. The resultant yeast strains were verified by PCR or sequencing.

#### Plasmids used for baculovirus generation and insect cell expression

All genes were cloned into the pFBDM transfer plasmid and the resultant plasmids are listed in Table S3. Plasmids were transformed into DH10Multibac cells and bacmids were isolated using isopropanol precipitation (Schreiber et al., 2011).

#### Protein expression in bacteria

For bacterial protein expression, plasmids listed in Table S3 were transformed into BL21-CodonPlus (DE3)-RIL cells (Agilent) unless stated otherwise. Cells were grown at 37°C in TB medium supplemented with ampicillin (100 μg/ml) and chloramphenicol (25 μg/ml). Cells were grown shaking at 220 rpm until they reached an OD_600_ of 0.8. Cells were moved on ice and protein expression was induced with 0.5 mM isopropyl-β-D-1-thiogalactopyranoside (IPTG). Protein expression was carried out overnight at 18°C. Cells were harvested at 4000 rpm for 10 minutes.

#### Protein expression in insect cells

Bacmids were prepared by isopropanol precipitation. GeneJuice was used to transfect Sf9 cells with bacmids. Viruses were further amplified using standard procedures. All proteins were expressed in High Five insect cells using Sf-900 II SFM medium. Cells were infected with a multiplicity of infection (MOI) greater than 2. Protein expression was carried out at 27°C with cells shaking at 140 rpm. Cells were harvested after three days.

#### Purification of Atg proteins and protein complexes

If not stated otherwise *S. cerevisiae* Atg proteins were purified at 4°C. Pre-cooled lysis buffer containing 50 mM Tris HCl pH 8.3, 300 mM NaCl (180 mM NaCl for protein complexes), 5% glycerol, 2 mM DTT, EDTA free protease inhibitor tablets (Roche), 2 mM EDTA, 0.2 mM PMSF, 1 mM benzamidine and Pierce universal nuclease was added to bacterial or insect cell pellets. The lysis buffer used for bacterial protein purifications was supplemented with lysozyme (100 μg/ml). Protease inhibitor tablets and irreversible protease inhibitors were omitted for the purification of enzymes with an active site cysteine. Instead PMSF (0.2 mM) leupeptin (10 μM), pepstatin A (10 μM) and EDTA (4 mM) were used. Cells were lysed by sonication and spun at 20,000 rpm for one hour using a JA-20 rotor.

#### Affinity purification

Supernatants were loaded onto a StrepTactin column (QIAGEN) or GST trap (GE Healthcare) pre-equilibrated with wash buffer composed of 50 mM Tris HCl pH 8.0, 300 mM NaCl (180 mM NaCl for protein complexes), 5% glycerol and 2 mM DTT. The column was washed with 10 column volumes (CV) wash buffer before proteins were eluted with 5 CV wash buffer containing either 2.5 mM desthiobiotin (StrepTactin) or 10 mM reduced glutathione (GST). Depending on the experiment, tags were cleaved overnight at 4°C using PreScission (3C) protease and a protease to protein molar ratio of 1:50.

#### Ion exchange chromatography

Protein containing afffinty purification fractions were pooled and diluted to a final salt concentration of 100 mM NaCl and subjected to ion exchange chromatography. Apart from Atg8 all proteins and protein complexes were purified by anion exchange chromatography using a ResQ column (GE Healthcare) applying a salt gradient from 50 to 700 mM NaCl (ResQ buffer base: 20 mM HEPES-NaOH pH 8.0, 5% glycerol and 2 mM DTT). Protein containing fractions were pooled, concentrated and either snap-frozen or further purified/analyzed by size exclusion chromatography. Proteins which were cleaved by PreScission protease were passed back over the initial affinity matrix in order to remove the tag or uncleaved protein.

#### Size exclusion chromatography

Samples were loaded on a size exclusion chromatography (SEC) column (Superose 6, Superdex 200 or Superdex 75 depending on the size of the protein/protein complex) pre-equilibrated in SEC buffer (20 mM HEPES NaOH pH 7.4, 180 mM NaCl, 5% glycerol and 2 mM DTT). Samples were concentrated using Amicon Ultra concentrators.

#### Atg8 purification

The affinity purification step was carried out as stated above. Tags were cleaved overnight using PreScission protease. StrepTactin elutions were diluted to a salt concentration of 30 mM NaCl using ResS Buffer A (20 mM MES pH 6.2, 5% glycerol and 2 mM DTT). The protein was loaded on a cation exchange column (ResS column; GE Healthcare) and eluted by applying a salt gradient from 30 to 500 mM NaCl (ResS buffer base: 20 mM MES pH 6.2, 5% glycerol and 2 mM DTT). The pH of the pooled peak fractions was adjusted to 8.0 and the sample was passed back over a 5 mL StrepTactin column. The StrepTactin column was washed with 1 CV of wash buffer and the flow-through and wash fractions were concentrated using Amicon concentrators (3 kDa cut off). The sample was run on a HiLoad 16/600 Superdex 75 column pre-equilibrated in SEC buffer (20 mM HEPES NaOH pH 7.4, 180 mM NaCl, 5% glycerol, 2 mM DTT). Peak fractions were concentrated and snap frozen.

#### Atg12–Atg5-Atg16 and Atg12^S^^>^^D_Ubl^–Atg5-Atg16 complex expression and purification

The Atg12–Atg5 or Atg12^S^^>^^D_Ubl^–Atg5 conjugate was produced in insect cells by co-expressing Atg5, Atg7, Atg10 and Atg12 or Atg12^S^^>^^D_Ubl^. To avoid truncation of Atg16 untagged or C-terminally FLAG-tagged Atg16 was expressed in BL21 RIL cells. Atg12–Atg5 was immobilized on a StrepTactin column prior to addition of sonicated bacterial cell lysates containing either Atg16 or Atg16-FLAG. The column was washed with 10 CV of wash buffer (50 mM Tris-HCl pH 8.0, 200 mM NaCl, 5% glycerol and 2 mM DTT) and the resultant E3 complex was eluted using 5 CV of elution buffer (wash buffer supplemented with 2.5 mM desthiobiotin). The E3 was further purified by anion exchange and size exclusion chromatography using a ResQ and Superose 6 column respectively (buffer composition as stated in the general protein purification protocol above). Note: The E3 was loaded on the ResQ column in elution buffer to avoid precipitation. The SEC run was carried out in SEC buffer containing 20 mM HEPES NaOH pH 7.4, 200 mM NaCl, 5% glycerol and 2 mM DTT.

#### Purification of full-length Atg9

Insect cells expressing full length Atg9 were lysed by passing the cell suspension through an EmulsiFlex. The lysate was cleared using a low speed spin (9000 g for 30 minutes), followed by centrifugation of the resulting supernatant at 40,000 rpm for 1 hour (Ti-45 rotor; Beckman). Pelleted membranes were resuspended in solubilization buffer (50 mM Tris HCl pH 8.0, 300 mM NaCl, 5% glycerol, 2 mM DTT) containing 1% n-Dodecyl-β-D-Maltopyranoside (DDM). The sample was incubated for 4 hours at 4°C before centrifuging at 40,000 rpm for 1 hour (Ti-45 rotor; Beckman). The supernatant was loaded onto a StrepTactin column pre-equilibrated in wash buffer (50 mM Tris HCl pH 8.0, 300 mM NaCl, 2 mM DTT containing either 0.6 mM DDM or 0.6 mM Lauryldimethylamine-N-Oxide (LDAO)). The column was washed with 10 CV of wash buffer and the protein was eluted using wash buffer containing 2.5 mM desthiobiotin. StrepTactin elutions were concentrated and used directly.

#### Purification of pre-phosphorylated Atg3

Atg3 was immobilized on a StrepTactin column (QIAGEN) and phosphorylated using recombinant Atg1 (100 nM). Phosphorylation reactions were carried out in 20 mM HEPES-NaOH pH 7.4, 180 mM NaCl, 2 mM ATP, 10 mM MgCl_2_ for 4 hours at 30°C. The phosphorylated E2 was eluted in StrepTactin elution buffer (50 mM Tris pH 8.0, 200 mM NaCl, 5% glycerol, 2 mM DTT, 2.5 mM desthiobiotin) and further purified using an anion exchange and size exclusion chromatography step (using a Resource Q and Superdex S75 column respectively). Fractions containing the phosphorylated E2 were pooled and concentrated. For direct control experiments the “non-phosphorylated” E2 was purified identically as the pre-phosphorylated E2, however, catalytically inactive Atg1 (Atg1^D211A^) was used instead of wild-type Atg1.

#### Purification of pre-phosphorylated Atg12–Atg5-Atg16

Atg12–Atg5-Atg16-FLAG was phosphorylated by incubating with wild-type Atg1 for 4 hours at 30°C. Phosphorylation reactions were carried out in 20 mM HEPES-NaOH pH 7.4, 180 mM NaCl, 2 mM ATP, 10 mM MgCl_2_ and 0.5X protein phosphatase inhibitors (Roche) using a kinase to substrate ratio of 1:50. Subsequently, the phosphorylated E3 (pE3) was immobilized using anti-FLAG M2 affinity resin (Sigma). The resin was washed four times with 15-20 bed volumes of wash buffer (20 mM HEPES pH 7.4, 300 mM NaCl, 5% glycerol, 2 mM DTT and 0.5X protein phosphatase inhibitors). The pE3 was eluted using wash buffer containing 100 μg/ml 3xFLAG peptide (Generon). FLAG elutions were diluted to 180 mM NaCl and the pE3 was further purified by ion exchange chromatography using a Resource Q column and a salt gradient ranging from 180-700 mM NaCl (ResQ buffer A: 20 mM HEPES-NaOH pH 7.4, 5% glycerol and 2 mM DTT and ResQ buffer B: 20 mM HEPES-NaOH pH 7.4, 700 mM NaCl, 5% glycerol and 2 mM DTT). Fractions containing pE3 were pooled and concentrated.

#### Purification of Tpk1 and Hrr25 kinases

SH-SUMO^∗^-Tpk1 was expressed in Rosetta 2(DE3) cells (Novagen) and SH-SUMO^∗^-Hrr25 was expressed in High Five insect cells. Both proteins were purified using a StrepTactin affinity purification step (see “Purification of Atg Proteins and Protein Complexes”). The SH-SUMO^∗^ tag was cleaved off overnight using GST-tagged PreScission protease. Kinases were further purified by SEC using a Superdex 200 column and kinase containing fractions were passed back over a StrepTactin and GST column before concentrating the proteins.

#### Purification of Sic1

SH-SUMO^∗^-Sic1 was expressed in Rosetta 2(DE3) cells and purified using a StrepTactin affinity purification step (see “Purification of Atg Proteins and Protein Complexes”). The SH-SUMO^∗^ tag was cleaved off overnight using PreScission protease. Sic1 was further purified by ion exchange chromatography using a Resource S column. Protein containing fractions were passed back over a StrepTactin column before being concentrated.

#### Purification of endogenous *S. cerevisiae* Atg3 and Atg12

Yeast strains expressing either wild-type or catalytically inactive Atg1 (Atg1^D211A^) and either endogenously SF-tagged Atg3 or Atg12 (yAS_476/yAS_538 and yAS_233/yASC_841 respectively) were grown in YPD medium in a fermenter. Cells were harvested at an OD_600_ of ∼1.0 and washed twice in nitrogen starvation (SD-N) medium. Cells were grown in SD-N medium for another 4 hours before harvesting. Pellets were resuspended in a small volume of resuspension buffer containing 50 mM Tris HCl pH 9.2, 170 mM NaCl, 5% glycerol, 2 mM DTT, 4 mM EDTA and protease and protein phosphatase inhibitors. Cell suspension was frozen in liquid nitrogen before freezer milling. Resuspension buffer supplemented with Pierce Universal Nuclease was added and the resultant lysate was spun at 20,000 rpm for one hour using a JA-20 rotor. Supernatants were loaded onto a pre-equilibrated 1 mL StrepTactin column (QIAGEN). The column was washed with 20 CV of wash buffer (50 mM Tris HCl pH 8.0, 180 mM NaCl, 5% glycerol, 2 mM DTT and protein phosphatase inhibitors). Samples were eluted with BXT buffer (IBA) containing 2 mM DTT (and 7 M UREA when preparing samples for mass spectrometry). Samples were either analyzed by Phos-Tag SDS-PAGE (50 μM Phos-tag acrylamide; 6% polyacrylamide gel; Alpha Laboratories) and Western blot analysis (Atg12) or further processed for phospho-enrichment and subsequent mass spectrometry analysis (Atg3). For the latter biological duplicates were prepared. The SF-tag in Atg3 was inserted between amino acids 266 and 268 as both N- and C-terminal tags impact autophagy (Ngu et al., 2015).

#### Fluorescent labeling of Atg8

N-terminally SF-tagged Atg8^M1C/ΔR117^ was expressed in bacteria and purified as stated above for wild-type Atg8. The SF-tag was cleaved using PreScission protease and Atg8 further purified by cation exchange chromatography (Buffer A: 20 mM HEPES-NaOH pH 7.0, 1% glycerol, 0.5 mM TCEP; Buffer B: 20 mM HEPES-NaOH pH 7.0, 700 mM NaCl, 1% glycerol, 0.5 mM TCEP). Atg8 was labeled by adding BODIPY TMR C_5_-Maleimide (Thermo Fisher) in 20-fold molar excess. The labeling reaction was incubated at 4°C overnight. Atg8 was separated from the dye by size exclusion chromatography using a Superdex 75 10/300 GL column (running buffer: 20 mM HEPES pH 7.4, 180 mM NaCl, 5% glycerol, 2 mM DTT).

#### *In vitro* kinase assays

Atg1 was pre-phosphorylated in the presence of 0.5 mM ATP, 2.5 mM MgCl_2_, 1 mg/ml BSA and 0.5X PhosSTOP protein phosphatase inhibitors (Roche). The final Atg1 concentration in the pre-phosphorylation reaction was 1 μM. Pre-phosphorylated Atg1 was diluted 20-fold resulting in a final assay concentration of 50 nM. Substrate phosphorylation was carried out in 20 mM HEPES pH 7.4, 150 mM NaCl, 425 μM ATP, 2.125 mM MgCl_2_, 7.5 μCi [γ-^32^P]-ATP (3000 Ci/mmol), 0.5 mg/ml BSA and 0.5X PhosSTOP protein phosphatase inhibitors (Roche). Substrates were used at a final assay concentration of 5 μM unless otherwise stated (e.g., the Vps34^Atg14^ and Vps34^Atg14/Atg38^ complex were used at 2.5 μM). Reactions were started by addition of pre-phosphorylated Atg1. Samples were taken at the indicated time points or after 10 minutes if no time point is specified. Phosphorylation reactions were stopped by addition of 4X SDS sample buffer containing 6 M UREA and 100 mM DTT. Kinase assays containing catalytically inactive Atg1 (Atg1^D211A^), the Vps34^Atg14/Atg38^ complex, Hrr25 or Tpk1 were carried out as detailed for wild-type Atg1. Peptides for AIM competition assays were purchased from GenScript, dissolved in DMSO and used at a final concentration of 300 μM. Myelin basic protein (MBP) was purchased from Lucerna-Chem.

#### Phos-Tag SDS-PAGE

Endogenous SF-tagged Atg12 purified from cells expressing either wild-type Atg1 or catalytically inactive Atg1^D211A^ was run on a 6% Phos-Tag SDS-PAGE gel containing 50 μM Phos-tag acrylamide (Alpha Laboratories) in 1x Tris glycine running buffer. The gel was washed twice in Protein Transfer Buffer containing 10 mM EDTA and once in EDTA free Transfer Buffer before western blotting using a PVDF membrane. SF-Atg12 was detected using a mouse anti-FLAG M2 antibody (Sigma) and goat anti-mouse IgG (H+L) HRP conjugate (Bio-Rad).

#### Atg8 lipidation assays

The following lipids were purchased from Avanti Polar Lipids: bovine L-α-phosphatidylethanolamine (PE; 840026C), bovine L-α-phosphatidylcholine (PC; 840055C), 1,2-dioleoyl-sn-glycero-3-phosphocholine (DOPC; 850375), bovine L-α-phosphatidylinositol (PI; 840042P) and brain L-α-phosphatidylserine (PS; 840032C). Lipids were dissolved in chloroform and mixed in the indicated ratios (wt%). Lipids were dried using nitrogen gas, washed using diethyl ether and dried again. The resultant lipid film was rehydrated in liposome resuspension buffer (20 mM HEPES pH 7.4, 100 mM NaCl), sonicated and subjected to three consecutive freeze-thaw cycles. Liposomes were sonicated prior to every downstream application. Lipidation assays were carried out in lipidation buffer (20 mM HEPES-NaOH pH 7.4, 120 mM NaCl, 10 mM MgCl_2_ and 2 mM ATP) using 5 μM Atg8, 1 μM Atg3, 0.5 μM Atg7 and 1 mg/ml liposomes unless otherwise stated. Reactions were started by addition of Atg7 or Atg8. Liposomes contained 55% PE, 35% PC and 10% PI for E3 independent lipidation assays and 25% PE, 45% PC, 5% PS and 25% PI for lipidation assays containing the E3. The latter contained the E3 at a final concentration of 2 μM. Lipidation reactions were incubated for the indicated time and stopped by addition of 6X Urea-SDS-PAGE loading buffer containing 100 mM DTT. Atg8 lipidation was analyzed by 15% Urea-SDS-PAGE (6 M urea). Gels were either stained by Coomassie or Sypro Ruby protein gel stain (Molecular Probes). Atg8 lipidation was quantified using Fiji.

To test the effect of Atg1 dependent phosphorylation on Atg8 lipidation in E3 independent Atg8 lipidation assays, Atg3/Atg7 and Atg8/Atg8^N^ were separately incubated with either wild-type Atg1 or Atg1^D211A^ (50 nM) and the lipidation reactions were started by combining the two pre-phosphorylation reactions.

In order to test the specificity of Atg1 mediated inhibition Atg3 and Atg7 were pre-phosphorylated using either wild-type Atg1, catalytically inactive Atg1 (Atg1^D211A^), Tpk1, Hrr25, the catalytic subunit of the cAMP-dependent protein kinase (PKA; NEB) or Plk1 (SignalChem).

To test the effect of Atg1 mediated phosphorylation on E3 ligase activity, the pre-phosphorylated E3 (pE3) was dephosphorylated using lambda protein phosphatase and PP2A^Rts1^. Dephosphorylation was carried out for 2 hours at 30°C. Reactions were stopped by addition of phosphatase inhibitors. Samples were moved on ice and Atg7 (0.5 μM), Atg3 (5 μM), liposomes (25% PE, 25% PI, 45% PC and 5% PS) and lipidation buffer were added. In order to generate a control sample, pE3 was handled identical to the dephosphorylation reaction, however, no protein phosphatases were added at this stage. Samples were moved on ice and Atg7, Atg3, liposomes and lipidation buffer were added (as specified above). A mix of lambda phosphatase, PP2A^Rts1^ and protein phosphatase inhibitors was added to the sample matching the protein phosphatase and protein phosphatase inhibitor concentration of the desphosphorylated E3 sample. Atg8 lipidation reactions containing either phosphorylated or dephosphorylated E3 were started by adding wild-type Atg8 (10 μM). Atg8 lipidation was monitored as a function of time and reactions were stopped by addition of 6X Urea sample buffer. Samples were analyzed by 15% Urea-SDS-PAGE.

#### Atg8 charging assays

Atg8∼Atg7 and Atg8∼Atg3 thioester formation was monitored by mixing Atg7 (0.5 μM) and Atg3 (5 μM) with TMR labeled Atg8^M1C^^/ΔR117^ (20 μM) in the presence of either 50 nM wild-type Atg1 or catalytically inactive Atg1^D211A^. Atg3/Atg7 and TMR-Atg8^M1C^^/ΔR117^ were separately incubated with either wild-type Atg1 or Atg1^D211A^ and the charging reactions were started by addition of TMR-Atg8^M1C^^/ΔR117^ to the Atg3/Atg7 mixture. Phosphorylation and charging reactions were incubated at 30°C in 20 mM HEPES-NaOH pH 7.4, 120 mM NaCl, 2 mM ATP, 10 mM MgCl_2_. Reactions were stopped by mixing 6 ul charging reaction with 8 ul 6X reducing agent free LDS sample buffer. Control samples were stopped by addition of 6X LDS loading dye containing 100 mM DTT. Samples were incubated at 50°C for 10 minutes and analyzed by SDS-PAGE. The resultant gels were imaged on a Typhoon scanner and subsequently stained with InstantBlue (Expedeon).

#### Atg3 discharge assays

Atg8, Atg3 and Atg7 were individually incubated with either 50 nM wild-type Atg1 or catalytically inactive Atg1^D211A^ before the charging reactions containing 10 μM Atg8, 5 μM Atg3-StrepII2x and 2 μM Atg7 were prepared. Atg3 charging was carried out in 8 mM HEPES-NaOH pH 7.4, 50 mM NaCl, 4 mM MgCl_2_ and 0.8 mM ATP. Samples were incubated for 30 minutes and EDTA was added to a final concentration of 50 mM. Reactions were started by addition of liposomes (1 mg/ml; containing 55% PE, 30% DOPC and 15% PI) and stopped by mixing 6 μL sample with 8 μL 6X reducing agent free LDS sample buffer. Control samples were taken at time point zero by adding 6X LDS loading dye containing 100 mM DTT. Samples were incubated at 50°C for 10 minutes and analyzed by SDS-PAGE and Coomassie staining.

#### Atg12 conjugation assays

Atg12 conjugation assays were carried out in lipidation buffer (20 mM HEPES-NaOH pH 7.4, 120 mM NaCl, 10 mM MgCl_2_ and 2 mM ATP) using 5 μM Atg12, 1 μM Atg10, 1 μM Atg7 and 20 μM Atg5-Atg16. Pre-phosphorylation reactions were carried out using 50 nM Atg1. Control reactions contained catalytically inactive Atg1^D211A^. Reactions were started by addition of Atg7, and stopped by addition of 6X Urea sample buffer. Samples were analyzed by SDS-PAGE.

#### Pho8Δ60 assays

“YPD” samples were prepared by growing yeast cells in nutrient-rich YPD medium at 30°C to an OD_600_ of ∼1.2. “SD-N” samples were prepared by switching yeast cells grown in YPD medium to starvation medium (SD-N). Cells were washed twice with SD-N medium before growing them for another 4 hours in SD-N medium (unless specified otherwise). Cells were harvested at 4000 rpm. Pellets were washed with ice-cold water, spun again and resuspended in resuspension solution (0.85% NaCl, 1 mM PMSF). Cells were processed as described previously (Klionsky, 2007) and alkaline phosphatase activity was measured using an end-point spectrophotometric assay monitoring hydrolysis of p-nitrophenolphosphate (pNPP) to p-nitrophenol (pNP). The average and standard deviations were calculated based on at least three biological replicates.

#### Pulldown assays

For Strep, Myc and FLAG pulldown experiments proteins were immobilized using either Strep-Tactin Superflow Plus (QIAGEN), Anti-c-Myc Agarose (Thermo Fisher) or Anti-FLAG M2 affinity resin (Sigma). Proteins were added and incubated with the resin for 15 minutes at room temperature. Beads were washed three times with 15-20 bed volumes each. Bound proteins were eluted using either 2.5 mM desthiobiotin (Sigma), 500 μg/ml Myc peptide (GenScript) or 100 μg/ml 3X FLAG peptide (Sigma) and analyzed by SDS-PAGE.

#### Liposome pelleting assays

Atg8 (10 μM) was lipidated as described above. Atg8 containing liposomes were pelleted and washed prior to sample addition. Proteins were incubated with Atg8 containing liposomes for 30 minutes. Liposomes were washed three times using wash buffer (20 mM HEPES-NaOH pH 7.4, 120 mM NaCl and 2 mM DTT). Liposomes were resuspended in Urea containing SDS sample buffer, and samples were analyzed by SDS-PAGE.

#### Fluorescence microscopy

For fluorescence microscopy experiments yeast cells were either exponentially grown in YPD medium or starved for 4 hours in nitrogen starvation medium (SD-N). Cells grown in YPD were pelleted and resuspended in synthetic complete (SC) medium prior to imaging. Images were acquired in a temperature-controlled environment (30°C) on an inverted wide-field Nikon Eclipse Ti microscope. Images were taken with a 100x oil objective. Atg proteins were imaged in the GFP channel and z stacks were recorded. Images were prepared using Fiji.

For co-localization experiments yeast strains yAS_610 and yASC_842 were transformed with the pRS425-tagBFP-Ape1 plasmid (Schütter et al., 2020; kindly provided by Martin Gräf), and grown to early log-phase in synthetic defined medium lacking leucine (SD-LEU) at 30°C. Cells were pelleted and resuspended in SD-LEU medium containing 250 μM CuSO_4_ and grown for another 3 hours. Cells were pelleted and washed 3 times in starvation medium (SD-N) and grown for 2 h 30 min in SD-N before imaging. The image datasets were acquired using a wide-field Nikon Ti2 inverted microscope, equipped with a 100X/1.45 NA lens and a Prime 95B sCMOS camera (Teledyne Photometrics), controlled through Micro-Manager 2.0 software (Edelstein et al., 2014). Z stacks of the BFP, neonGreen, and transmitted light channels were acquired using selective band-pass filters, over a range of 10 μm, every 0.25 μm. Deconvolution of the fluorescence channels was obtained processing the datasets with scikit-image 0.18.1 python package (van der Walt et al., 2014), adopting the Richardson-Lucy algorithm, with 15 iterations.

#### Fluorescence recovery after photobleaching (FRAP) experiments

Atg11 deleted yeast expressing Atg13-neonGreen and either wild-type Atg1 or catalytically inactive Atg1^D211A^ (yAS_621 and yAS_554 respectively) were grown in YPD medium and switched to nitrogen starvation medium (SD-N). Cells were grown in SD-N medium for 3 hours before they were imaged using a Leica TSC SP8 confocal microscope. GFP was excited with an argon laser at 488 nm, and emission was recorded between 498 nm–758 nm. Cells were imaged with a 63 × /1.40 oil objective and images were acquired every 10 s for one minute after photobleaching. Images for each time point were bleach corrected and the fluorescence intensity of the bleached area was compared to the initial intensity after background subtraction.

#### Mass spectrometry

After *in vitro* phosphorylating Atg proteins (2 μM) with Atg1 (50 nM), samples were TCA precipitated and the resultant pellets washed twice with ice-cold acetone and resuspended in ABC urea buffer (50 mM NH_4_HCO_3_, 8 M Urea). TCEP was added to a final concentration of 5 mM. After 30 minutes at room temperature, iodoacetamide was added to a final concentration of 10 mM and reactions were incubated for another 30 minutes in the dark. Samples were diluted to a final urea concentration of 6 M using ABC buffer (50 mM NH_4_HCO_3_)), before incubating for 4-5 hours at 37°C with Lysyl-endopeptidase (LysC; Wako) (1:100). Reactions were diluted to 2 M Urea and incubated with trypsin overnight shaking in the dark. Formic acid (FA) was added to a final concentration of 1% and samples were loaded on a pre-equilibrated C18 Sep-PAK column (Waters). Columns were washed with 2% acetonitrile (ACN) containing 0.1% FA and samples were eluted with 50% ACN containing 0.1% FA. Samples were dried in a SpeedVac, resuspended in 5% ACN containing 0.1% FA, sonicated and analyzed by mass spectrometry. The same procedure was followed for endogenous Atg3 and Atg12 containing samples with the only differences that samples were not TCA precipitated and only trypsin was used for protein digestion. C18 cleaned samples were phospho-enriched using the High-Select Fe-NTA Phosphopeptide Enrichment Kit (Thermo Fisher Scientific) following the manufacturer’s instructions.

Mass spectrometry data were acquired in data dependent acquisition mode using a 5600 TripleTOF (Sciex) or LTQ-Orbitrap XL (Thermo Fisher Scientific) instrument for *in vitro* kinase reactions, or a TimsTOF Pro for endogenously purified Atg3 samples. For TripleTOF acquisition peptides were separated using an Eksigent NanoLC Ultra nanoLC system using a 60 min gradient from 2%–35% (buffer A 0.1% (v/v) formic acid, 2% (v/v) acetonitrile, buffer B 0.1% (v/v) formic acid, 90% (v/v) acetonitrile) after direct injection onto a 20-cm PicoFrit emitter (New Objective) packed to 20 cm with Magic C18 AQ 3-μm 200-Å stationary phase. MS1 spectra were collected for 250 ms with nominal resolving power of 30,000. The 20 most intense precursors with charge state 2–5 were selected for fragmentation, and MS2 spectra were collected in the range 50–2,000 m/z for 100 ms with nominal resolving power of 15,000 (high sensitivity mode); precursor ions were excluded from reselection for 15 s. For LTQ-Orbitrap XL acquisition peptide separation was carried out by reversed phase on a Proxeon EASY-nLC II liquid chromatography system (Thermo Fisher Scientific). The reverse phase column (75 mm x 10 cm) was packed with Magic C18 AQ 3-μm 200-Å stationary phase. A linear gradient from 5% to 35% acetonitrile in 0.1% formic acid was run for 60 min at a flow rate of 300 nL/min. Data acquisition was set to obtain one high resolution MS scan in the Orbitrap (60,000 at 400 m/z) followed by six collision induced fragmentation (CID) MS/MS fragment ion spectra in the linear trap quadrupole (LTQ). Orbitrap charge state screening was enabled and ions with unassigned or single charge states were rejected. The dynamic exclusion window was set to 15 s and limited to 300 entries. The minimal precursor ion current to trigger CID and MS/MS scan was set to 150. The ion accumulation time was set to 500 ms (MS) and 250 ms (MS/MS) using a target setting of 10^6^ (MS) and 10^4^ (MS/MS) ions. For TimsTOF Pro data acquisition peptide separation was carried on a nanoElute liquid chromatography system (Bruker) using a reversed phase nanoElute TEN (Bruker) 75mm x 10 cm column packed with 1.9 μm C18 ReproSil AQ beads maintained at 50°C using a linear gradient from 3% to 28% acetonitrile in 0.1% formic acid at 400 nl/min over 60 minutes. Data was acquired in ddaPASEF mode (Meier et al., 2018) using the vendor provided standard 1.1 s cycle time acquisition method (10 PASEF ramps per cycle, collision energy ramp 20-59 V).

MS/MS spectra were searched in MaxQuant v1.6.12.0 (Cox and Mann, 2008) against a yeast protein sequence database retrieved from UniProt (September 2020 - strain ATCC 204508 / S288c – taxon identifier – 559292 - containing 6164 sequences) appended with common contaminants, affinity tag sequences, and decoys generated by protein sequence reversal. Search settings were set to fully-tryptic cleavage with 2 missed cleavages allowed, variable modification of oxidation allowed on methionine, phosphorylation on serine/threonine/tyrosine, acetylation on protein N terminus and fixed modification of carbamidomethylation on cysteines. All other search parameters were set to default for the respective instruments. False discovery rate was controlled at 1% at peptide and protein levels. For quantification we extracted MS1 level extracted Ion chromatograms using Skyline v20.2.1.404. Mapping of peptide identifications and phosphosite localizations was performed by importing MaxQuant search results into Skyline.

### Quantification and statistical analysis

As indicated in the figure legends data are represented as average ± standard deviation.

## Data Availability

• The mass spectrometry data have been deposited to the ProteomeXchange Consortium via the PRIDE (Perez-Riverol et al., 2019) partner repository and are publicly available. The accession number is listed in the key resources table. All other data are provided in the manuscript and supplemental information. • This paper does not report original code. • Any additional information required to reanalyze the data reported in this paper is available from the lead contact upon request.

## Supporting citation

The following reference appears in the supplemental information: Huh et al., 2003.

## Supplemental information

Document S1. Figures S1–S6 and Tables S2 and S3

Table S1. *In vitro* and *in vivo* Atg1-dependent phosphorylation sites, related to Figures 1, 2, 3, 4, and 5

Table S4. DNA oligonucleotides used in this study, related to STAR Methods

Document S2. Article plus supplemental information
