# Supplementary material for: Multilayered regulation of autophagy by the Atg1 kinase orchestrates spatial and temporal control of autophagosome formation
Source: Mol Cell. 2021 Dec 16;81(24):5066–5081.e10. doi: 10.1016/j.molcel.2021.10.024 (PMC8693860; doi:10.1016/j.molcel.2021.10.024)
Supplement: Document S1. Figures S1–S6 and Tables S2 and S3 [file mmc1.pdf]

Molecular Cell, Volume *81*

Supplemental information

Multilayered regulation of autophagy by the Atg1 kinase orchestrates spatial and temporal control of autophagosome formation

Anne Schreiber, Ben C. Collins, Colin Davis, Radoslav I. Enchev, Angie Sedra, Rocco D'Antuono, Ruedi Aebersold, and Matthias Peter

Supplementary Figure 1. *In vitro* specificity of Atg1 mediated substrate phosphorylation.
Related to Figure 1

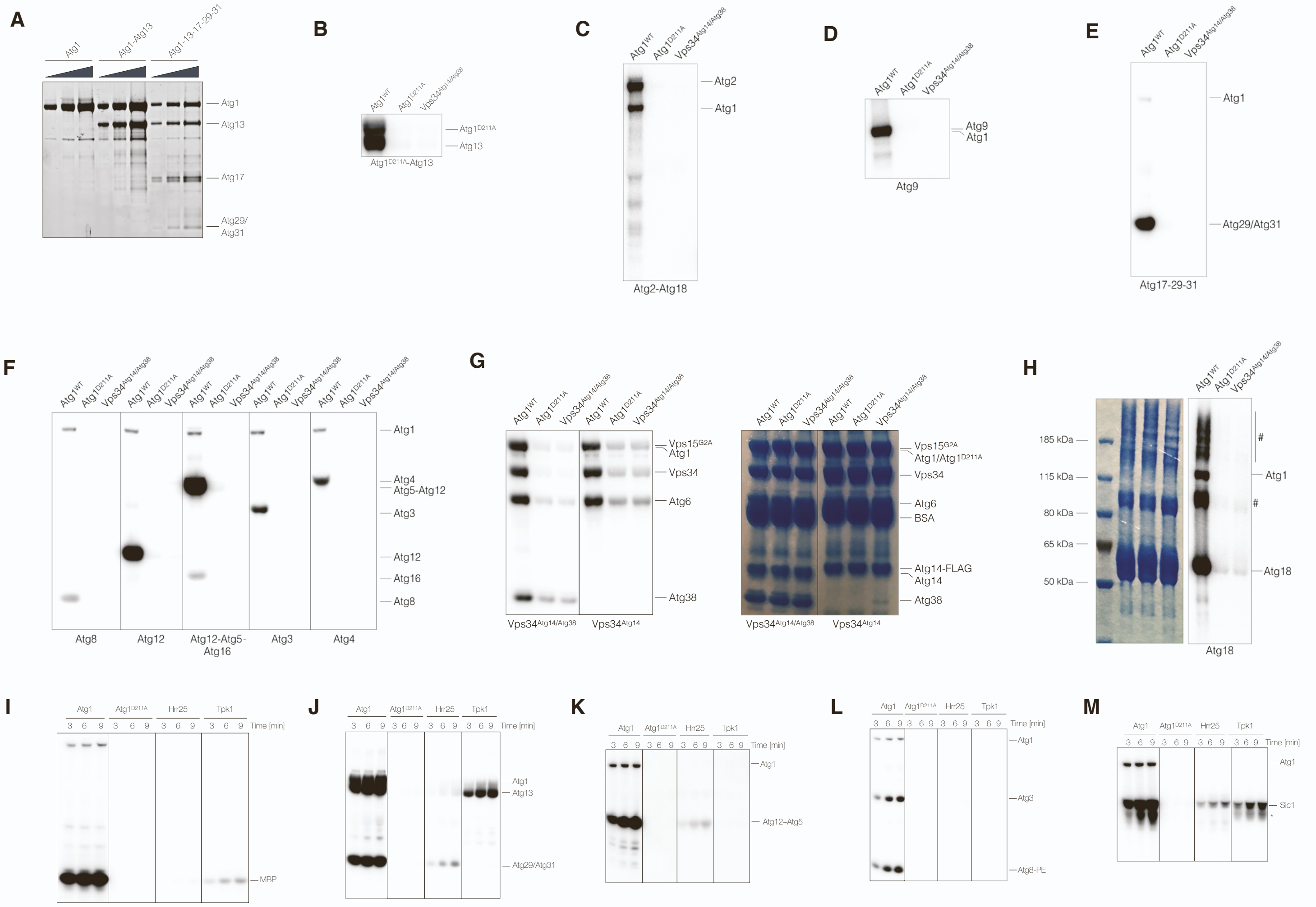

Supplementary Figure 1. *In vitro* specificity of Atg1 mediated substrate phosphorylation.

A) SDS-PAGE analysis of recombinant Atg1 preparations used in this study. Atg1 was purified either alone, in complex with Atg13, or as part of a complex containing Atg13, Atg17, Atg29 and Atg31. Increasing amounts of the three different Atg1 preparations were analyzed by SDS-PAGE and Sypro Ruby staining. (**B-H**) Phosphorylation of core Atg proteins was monitored in the presence of wild type Atg1, catalytically inactive Atg1 (Atg1^{D211A}) or the autophagosomal Vps34$^{Atg14/Atg38}$ complex, which contains the putative protein kinase Vps15. The following substrates were tested in the *in vitro* kinase assays using radioactively labelled [γ-^{32}P]-ATP: (**B**) catalytically inactive Atg1^{D211A}-Atg13, (**C**) the Atg2-Atg18 complex, (**D**) the transmembrane protein Atg9, (**E**) the Atg17-Atg29-Atg31 subcomplex, (**F**) Atg8, Atg12, Atg12–Atg5-Atg16, Atg3 and Atg4, (**G**) the tetrameric autophagosomal Vps34^{Atg14} complex comprising StrepII^{2x}-Vps15^{G2A}, Vps34, Atg6 and Atg14-FLAG and the pentameric Vps34$^{Atg14/Atg38}$ complex comprised of StrepII^{2x}-Vps15^{G2A}, Vps34, Atg6, Atg14 and Atg38, and (**H**) Atg18. (**I-M**) Phosphorylation of Atg proteins (5 μM) was monitored in the presence of wild type Atg1, catalytically inactive Atg1^{D211A}, Hrr25 or Tpk1. All kinases were incubated with ATP/Mg^{2+} prior to substrate addition to allow for autophosphorylation. *In vitro* kinase assays were started by addition of pre-phosphorylated Atg1 (50 nM final concentration). Time points were analysed by SDS-PAGE and autoradiography. The following substrates were tested: (**I**) the generic protein kinase substrate myelin basic chain protein (MBP), (**J**) the catalytically inactive Atg1^{D211A}-Atg13-Atg17-Atg29-Atg31 complex, (**K**) the E3 ligase Atg12–Atg5-Atg16, (**L**) Atg8-PE and Atg3 (reactions also contained 0.5 μM Atg7 to catalyse Atg8 lipidation prior to Atg1 addition) and (**M**) Sic1, a highly phosphorylated *S. cerevisiae* protein with no known relevance for autophagy (degradation products are marked with an asterisk).

Supplementary Figure 2. Atg1 kinase activity is stimulated by Atg8-PE.
Related to Figure 2

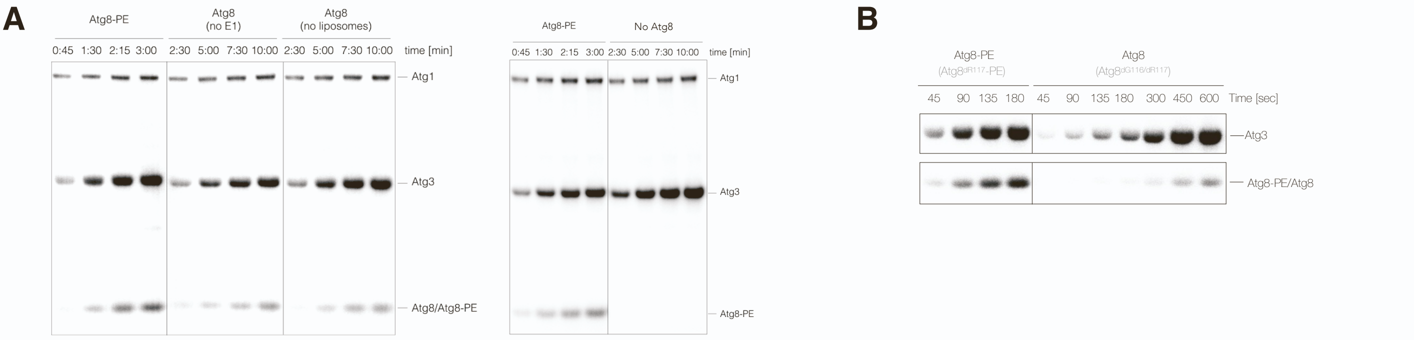

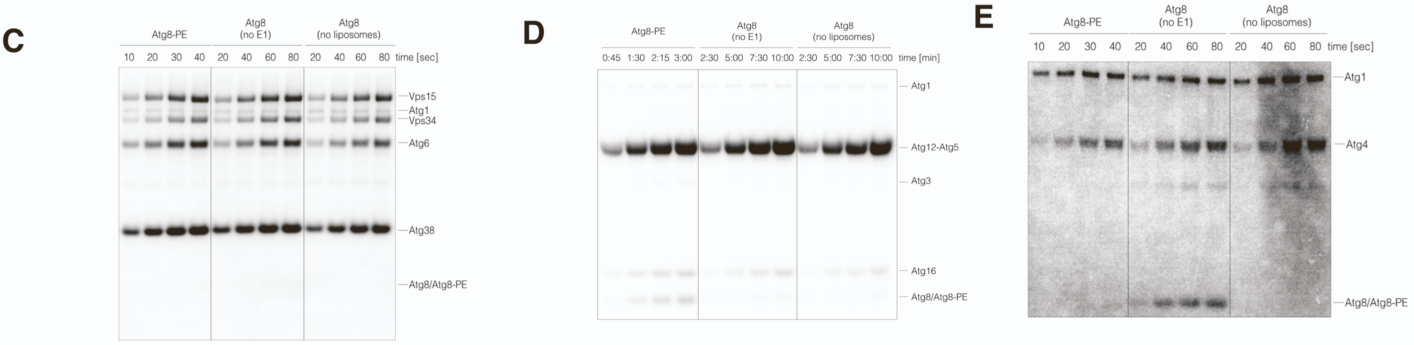

Relative Phosphorylation Rates [a.u.]
Substrate: $Vps34^{Atg14/Atg38}$

	Vps15	Vps34	Atg6	Atg38
Atg8-PE	26.5 +/- 5.7	13.9 +/-3.5	28.8 +/- 6.5	64.7 +/- 12.4
Atg8 (No Atg7)	13.8 +/- 1.4	8.5 +/- 1.4	13.4 +/- 2.9	27.4 +/- 3.8
Atg8 (No liposomes)	9.0 +/- 0.3	6.3 +/- 0.1	11.8 +/- 0.6	19.5 +/- 3.4

Relative Phosphorylation Rates [a.u.]
Substrate: Atg12-Atg5/Atg16

	Atg16	Atg5-12
Atg8-PE	5.97 +/- 1.15	19.71 +/- 3.23
Atg8 (No Atg7)	1.91 +/- 0.21	7.19 +/- 0.97
Atg8 (No liposomes)	1.57 +/- 0.04	5.69 +/- 0.18

Relative Phosphorylation Rates [a.u.]
Substrate: $Atg4^{C147A}$

	$Atg4^{C147A}$
Atg8-PE	1.35 +/- 0.45
Atg8 (No Atg7)	0.72 +/- 0.04
Atg8 (No liposomes)	0.63 +/- 0.11

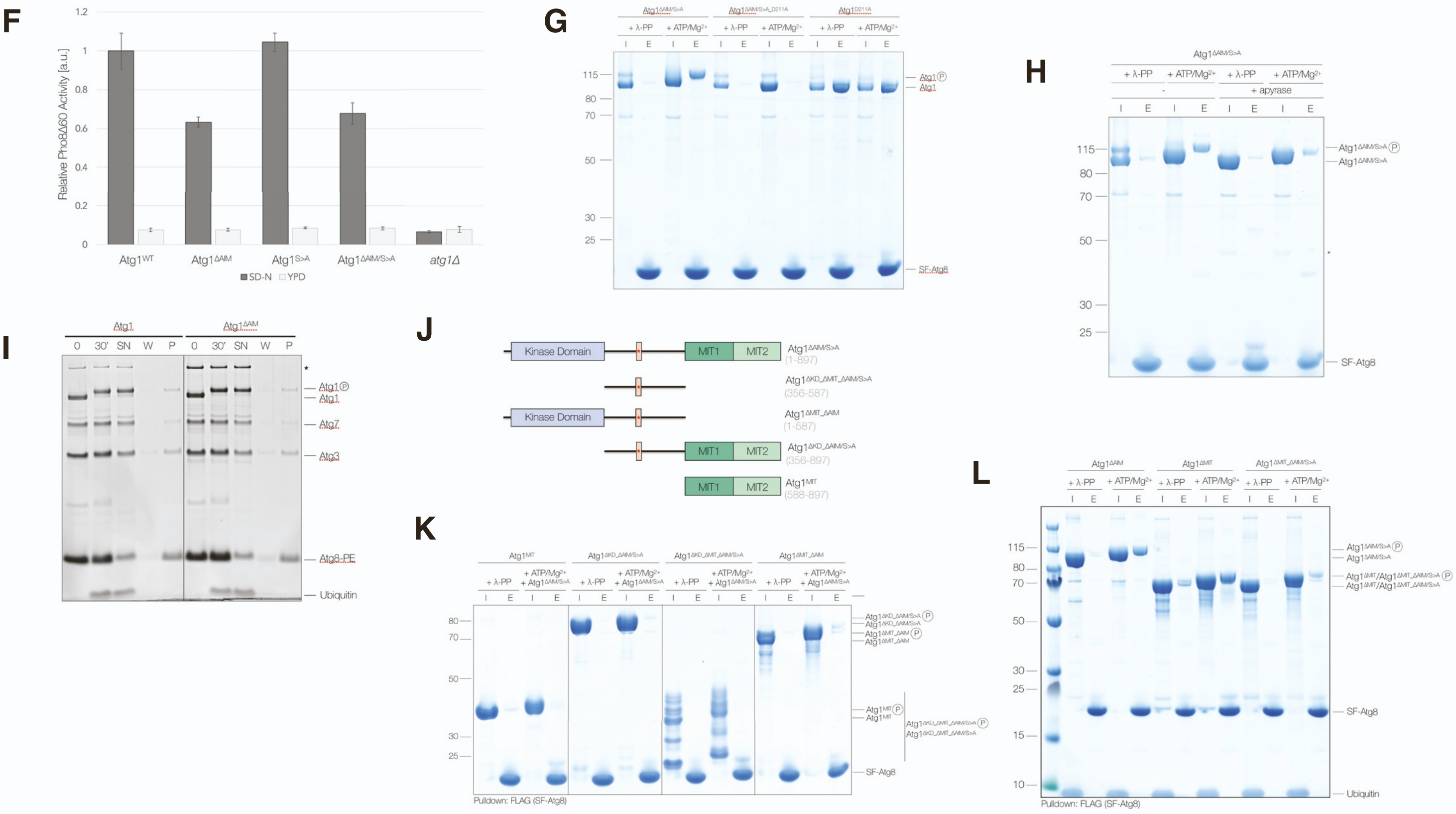

Supplementary Figure 2. Atg1 kinase activity is stimulated by Atg8-PE.

A) Atg1 dependent phosphorylation of Atg3 and Atg8/Atg8-PE in the presence of either Atg8 or lipidated Atg8-PE. Atg8 was lipidated prior to starting *in vitro* kinase assays by addition of pre-phosphorylated Atg1. Control reactions lacked either Atg7 (no E1), liposomes or Atg8. Atg8/Atg8-PE and Atg3 phosphorylation was monitored in a time dependent manner using radioactively labelled [γ-^{32}P]-ATP. Time points were analyzed by SDS-PAGE and autoradiography. Note: different time points were used for Atg8 and Atg8-PE containing reactions. Data quantification is shown in Figure 2C. **B**) Atg1 dependent phosphorylation of Atg3 and Atg8/Atg8-PE in the presence of either soluble Atg8 (Atg8$^{\Delta G116/\Delta R117}$) or lipidated Atg8 (Atg8$^{\Delta R117}$-PE). Prior to starting the *in vitro* kinase reactions by adding pre-phosphorylated Atg1, Atg8$^{\Delta G116/\Delta R117}$ and Atg8$^{\Delta R117}$ were incubated with recombinant Atg7, Atg3 and PE containing liposomes (55% PE, 35% PC and 10% PI) to promote complete Atg8$^{\Delta R117}$ lipidation (left panel). The Atg8$^{\Delta G116/\Delta R117}$ mutant binds Atg1 but cannot be lipidated therefore acting as a control (right panel). The Atg3 concentration was adjusted to 5 µM prior to starting *in vitro* kinase reactions by adding pre-phosphorylated Atg1 (50 nM). Substrate (5 µM) phosphorylation was analysed at indicated time points by SDS-PAGE and autoradiography. (**C-E**) Atg1 dependent phosphorylation of different Atg1 substrates in the presence of either Atg8-PE or Atg8. Atg8 was lipidated prior to substrate addition. The substrate concentration was 2 µM with the exception of the Vps34$^{Atg14/Atg38}$ complex which was used at 1 µM. Phosphorylation reactions were started by adding pre-phosphorylated Atg1 (50 nM). Substrate phosphorylation was studied in the presence of 0.5 µM Atg7, 0.5 µM Atg3, 5 µM Atg8/Atg8-PE and liposomes containing 55% PE, 35% PC and 10% PI. Control reactions either lacked the E1 Atg7 or liposomes. Relative phosphorylation rates were quantified (n=3) and listed as arbitrary units [a.u.] in the table below. The following Atg1 substrates were tested: (**C**) the pentameric autophagosomal Vps34$^{Atg14/Atg38}$ complex, (**D**) the Atg12–Atg5-Atg16 complex and (**E**) the Atg8 specific protease Atg4 (an active site mutant of Atg4, Atg4^{C147A}, was used in all reactions to prevent delipidation of Atg8 in reactions containing Atg8-PE). **F**) The Pho8Δ60 assay was used to quantify bulk autophagy in *atg1Δ* strains or in cells containing either wild type Atg1 (Atg1^{WT}), Atg1$^{Y429A/V432A}$ (Atg1$^{\Delta AIM}$), Atg1^{S418A_S421A_S424A} (Atg1$^{S>A}$) or Atg1$^{S418A/S421A/S424A/Y429A/V432A}$ (Atg1$^{\Delta AIM/S>A}$). Cells were either exponentially grown in YPD medium or starved for 4 hours in starvation medium (SD-N). Alkaline phosphatase activity was measured (n=3) and plotted as relative Pho8Δ60 activity in arbitrary units (a.u.) with corresponding standard deviation. **G)** Atg1 pulldown experiments were carried out using SF-tagged Atg8 (SF-Atg8) as bait. A catalytically active and inactive Atg1 AIM mutant (Atg1$^{\Delta AIM/S>A}$ and Atg1$^{\Delta AIM/S>A_D211A}$ respectively) and catalytically inactive Atg1^{D211A} were incubated either with ATP/Mg^{2+} or lambda protein phosphatase (λ-PP) before addition to SF-Atg8 containing anti-FLAG M2 resin. SF-Atg8 and co-purifying proteins were eluted using 3X FLAG peptide and the resultant elutions (E) were analyzed by SDS-PAGE and Coomassie staining. **H**) Pulldown experiments using SF-Atg8 as a bait. Binding of Atg1$^{\Delta AIM/S>A}$ to phosphorylated (lanes 1-4) or non-phosphorylated Atg8 (lanes 5-8) was compared. Atg1$^{\Delta AIM/S>A}$ was either phosphorylated (ATP/Mg^{2+}) or dephosphorylated using λ-PP before the input samples (I) were added to SF-Atg8 containing anti-FLAG M2 resin. To prevent phosphorylation of Atg8 apyrase was added for 1 hour to one set of reactions prior to incubation with SF-Atg8 containing anti-FLAG M2 resin. SF-Atg8 and co-purifying proteins were eluted with 3X FLAG peptide. The resultant elutions (E) were analyzed by SDS-PAGE and Coomassie staining. **I)** Liposome pelleting assays comparing Atg8-PE binding of wild type Atg1 and an Atg1 AIM mutant (Atg1$^{\Delta AIM}$). Atg1 and Atg1$^{\Delta AIM}$ were added to Atg8-PE containing liposomes (0') and phosphorylated for 30 minutes (30'). Ubiquitin was added as a specificity control before liposomes were pelleted. The supernatant (SN) was removed, and liposomes were washed (W) three times. The final pellet was resuspended in SDS loading dye (P) and analyzed by SDS-PAGE and Sypro Ruby staining. Note: Atg8 containing liposomes also contained Atg3 and Atg7. **J)** Summary of Atg1 truncation mutants used to study Atg8 binding. The red box with asterisk indicates the previously reported AIM, which was mutated to alanine in all constructs containing the central region. (**K-L)** Pulldown experiments using SF-Atg8 as a bait. Atg8 binding was compared between different Atg1 AIM and Atg1 truncation mutants. Atg1 preparations were either autophosphorylated (ATP/Mg^{2+}) or dephosphorylated using λ-PP before addition to SF-Atg8 coated anti-FLAG M2 resin. Atg8 and co-purifying proteins were eluted with 3X FLAG peptide and the resultant elutions (E) were analyzed by SDS-PAGE and Coomassie staining. The following constructs were tested in pulldown experiments: (**K**) the Atg1 MIT domain (Atg1^{MIT}), SH-SUMO*-Atg1$^{\Delta KD_\Delta AIM/S>A}$, SH-SUMO*-Atg1$^{\Delta KD_\Delta MIT_\Delta AIM/S>A}$ and StrepII^{2x}-Atg1$^{\Delta MIT_\Delta AIM}$ and (**L**) StrepII^{2x}-Atg1$^{\Delta AIM}$, StrepII^{2x}-Atg1$^{\Delta MIT}$ and StrepII^{2x}-Atg1$^{\Delta MIT_\Delta AIM/S>A}$. Substoichiometric amounts of StrepII^{2x}-Atg1$^{\Delta AIM/S>A}$ (1:20) were used to phosphorylate all constructs.

Supplementary Figure 3. Atg1 complex disassembly is triggered by Atg1 dependent phosphorylation of Atg13.

Related to Figure 3

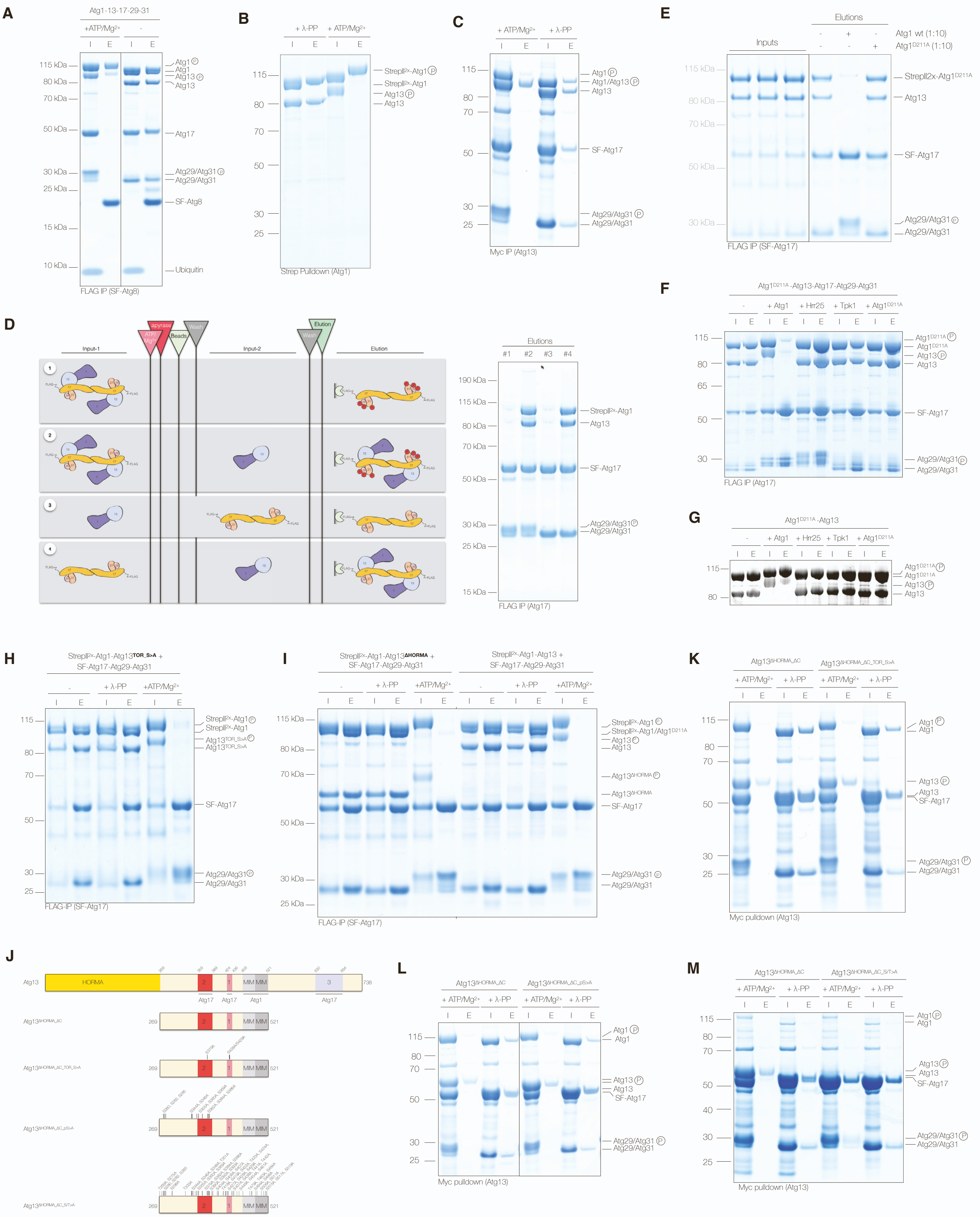

Supplementary Figure 3. Atg1 complex disassembly is triggered by Atg1 dependent phosphorylation of Atg13.

A) The Atg1-Atg13-Atg17-Atg29-Atg31 complex was incubated with or without ATP/Mg^{2+}. Samples were mixed with ubiquitin (I: Input) and added to beads containing StrepII^{2x}-FLAG-tagged Atg8 (SF-Atg8). Ubiquitin was used as a specificity control. SF-Atg8 and co-purifying proteins were eluted (E) and analyzed by SDS-PAGE and Coomassie staining. **B)** The StrepII^{2x}-Atg1-Atg13 complex was incubated with or without ATP/Mg^{2+} (I: Input) and subsequently immobilised using StrepTactin resin. StrepTactin elutions (E) were analysed by SDS-PAGE and Coomassie staining. **C**) Myc-tagged Atg13 (StrepII^{2x}-Atg13-Myc) was combined with Atg1 and the Atg17-Atg29-Atg31 subcomplex and either incubated with ATP/Mg^{2+} or lambda protein phosphatase (λ-PP). Atg13 was immobilized using anti-c-Myc agarose. Atg13 and co-purifying proteins were eluted and analyzed by SDS-PAGE and Coomassie staining. **D)** Schematic representation (left) and elutions (right) of pulldown experiments probing protein-protein interactions sensitive to Atg1 mediated phosphorylation. The Atg1-Atg13, Atg1-Atg13-Atg17-Atg29-Atg31 and Atg17-Atg29-Atg31 complexes were incubated with ATP/Mg^{2+} (Input-1). Phosphorylation was stopped by ATP depletion using apyrase. Anti-FLAG M2 resin was added, washed (with the exception of reaction 3) and samples 2 and 4 were mixed with Atg1-Atg13 while sample 3 was incubated with SF-Atg17-Atg29-Atg31 (Input-2). Resins were washed again and proteins co-purifying wit SF-Atg17 were eluted and analyzed by SDS-PAGE and Coomassie staining. **E)** Substoichiometric amounts (1:10) of wild type Atg1 or catalytically inactive Atg1 (Atg1^{D211A}) were added to the catalytically inactive Atg1^{D211A}-Atg13-Atg17-Atg29-Atg31 complex in the presence of ATP/Mg^{2+}. SF-Atg17 was immobilized using anti-FLAG M2 resin and SF-Atg17 and co-purifying proteins were eluted and analyzed by SDS-PAGE and Coomassie staining. **F**) The catalytically inactive Atg1^{D211A}-Atg13-Atg17-Atg29-Atg31 complex was incubated with substoichiometric amounts of Atg1, Hrr25, Tpk1 or catalytically inactive Atg1^{D211A} (1:20) in the presence of ATP/Mg^{2+}. Phosphorylation reactions (I) were added to anti-FLAG M2 agarose and proteins co-purifying with SF-Atg17 were analyzed by SDS-PAGE and Coomassie staining. **G**) The catalytically inactive Atg1^{D211A} -Atg13 complex was incubated with substoichiometric amounts of Atg1, Hrr25, Tpk1 or catalytically inactive Atg1^{D211A} (1:20) in the presence of ATP/Mg^{2+} prior to immobilising StrepII^{2x}-tagged Atg1 using StrepTactin sepharose. Proteins were eluted and analysed by SDS-PAGE and Sypro Ruby staining. **H)** An Atg1 complex containing an Atg13 mutant with the three TORC1 phosphorylation sites known to regulate Atg17 binding mutated to alanine (Atg1-Atg13$^{TOR_S>A}$-Atg17-Atg29-Atg31) was incubated with ATP/Mg^{2+} or λ-PP. Reactions (I: Input) were subjected to FLAG immunoprecipitation (IP) and proteins co-purifying with SF-Atg17 (E: Elution) were analyzed by SDS-PAGE and Coomassie staining. Atg13$^{TOR_S>A}$: Atg13$^{S379A/S428A/S429A}$. **I)** An Atg1 complex lacking the Atg13 HORMA domain (Atg1-Atg13$^{\Delta HORMA}$-Atg17-Atg29-Atg31) was incubated with ATP/Mg^{2+} or λ-PP. Reactions (I: Input) were added to anti-FLAG M2 agarose and SF-Atg17 and co-purifying proteins were eluted and analyzed by SDS-PAGE and Coomassie staining. **J**) Domain overview of *S. cerevisiae* Atg13 highlighting the N-terminal HORMA domain, the three Atg17 binding sites and the two MIT-interacting motifs (MIMs) which mediate Atg1 binding. Constructs used in this study are shown below, with amino acids mutated to alanine indicated. All Atg13 constructs lacking the HORMA domain and the C-terminal region (Atg13$^{\Delta HORMA_\Delta C}$) have an N-terminal SH-SUMO*-tag and a C-terminal Myc tag. (**K-M**) The following Myc-tagged Atg13 constructs were mixed with Atg1 and the Atg17-Atg29-Atg31 complex. Reactions were either phosphorylated (ATP/Mg^{2+}) or dephosphorylated using λ-PP. Reactions (I) were added to anti-c-Myc agarose and Myc-tagged Atg13 constructs and co-purifying proteins were eluted (E) and analyzed by SDS-PAGE and Coomassie staining. (**K**) Atg13$^{\Delta HORMA_\Delta C}$ and Atg13$^{\Delta HORMA_\Delta C_TOR_S>A}$, (**L**) Atg13$^{\Delta HORMA_\Delta C}$ and Atg13$^{\Delta HORMA_\Delta C_pS>A}$ and (**M**) Atg13$^{\Delta HORMA_\Delta C}$ and Atg13$^{\Delta HORMA_\Delta C_S/T>A}$. Note: only substoichiometric amounts of Atg1 were used.

Supplementary Figure 4. Phosphorylation of the Atg13 central region triggers Atg1-Atg13 complex dissociation.
Related to Figure 3

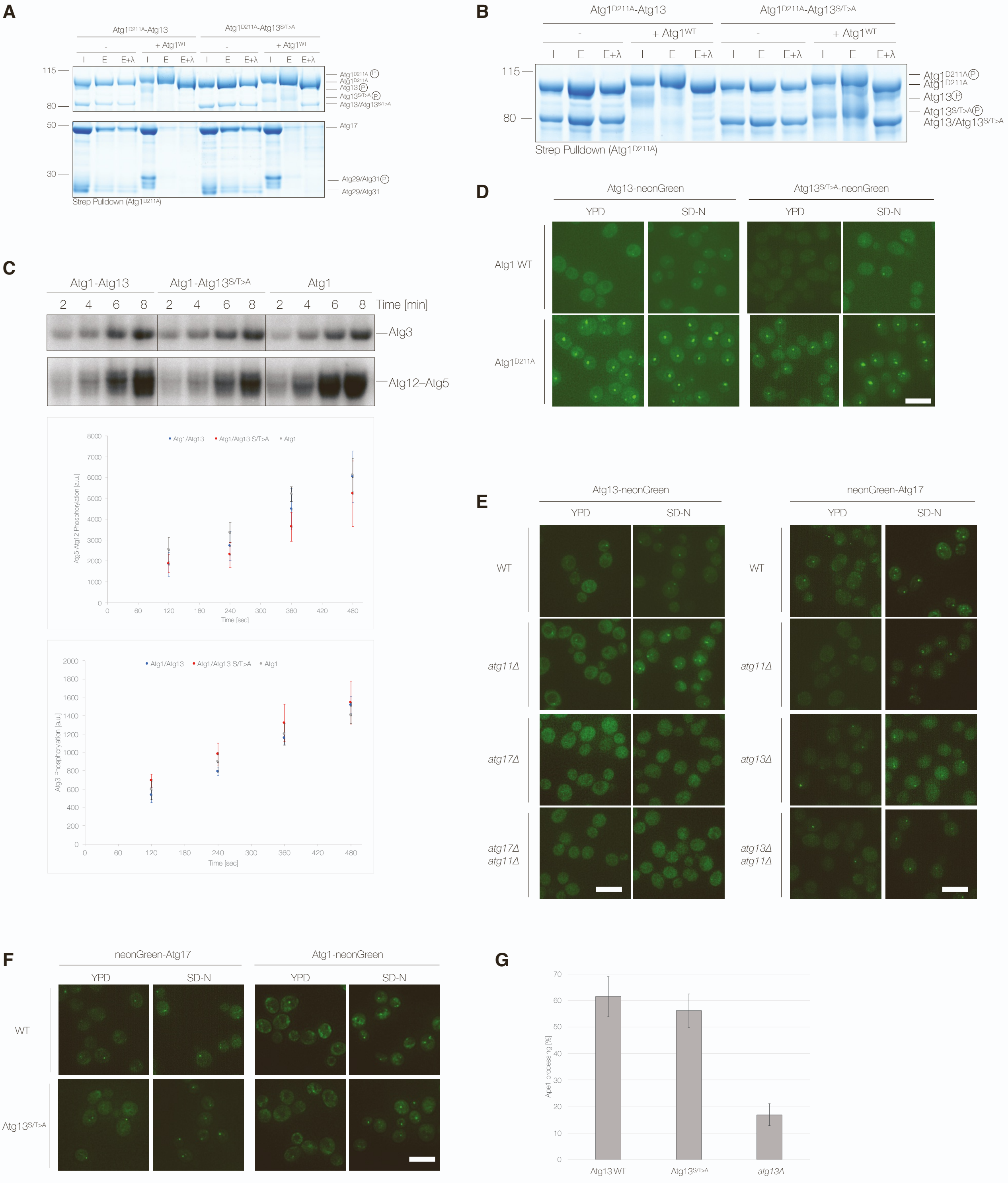

Supplementary Figure 4. Phosphorylation of the Atg13 central region triggers Atg1-Atg13 complex dissociation.

A) The Atg1^{D211A}-Atg13 and Atg1^{D211A}-Atg13$^{S/T>A}$ complex were incubated with the Atg17-Atg29-Atg31 subcomplex and with or without wild type Atg1 in the presence of ATP/Mg^{2+} (I). Complexes were immobilized using StrepTactin Sepharose and StrepII^{2x}-Atg1^{D211A} and co-purifying proteins were eluted (E) and subsequently incubated with λ-PP (E+λ) prior to analysis by SDS-PAGE and Coomassie staining. **B)** The Atg13$^{S/T>A}$ mutant does not dissociate from Atg1^{D211A} upon Atg1 mediated phosphorylation. The Atg1^{D211A}-Atg13 and Atg1^{D211A}-Atg13$^{S/T>A}$ complex were incubated with or without substoichiometric amounts of wild type Atg1 in the presence of ATP/Mg^{2+} (I). Complexes were immobilized using StrepTactin Sepharose and StrepII^{2x}-Atg1^{D211A} and co-purifying proteins were eluted (E) and subsequently incubated with λ-PP (E+λ) prior to analysis by SDS-PAGE and Coomassie staining. **C)** *In vitro* kinase assays studying phosphorylation of Atg3 and Atg12–Atg5-Atg16 (5 μM) by Atg1, Atg1-Atg13 or Atg1-Atg13$^{S/T>A}$ (50 nM). Substrate phosphorylation was studied in a time dependent manner and individual time points were analysed by SDS-PAGE and autoradiography. Relative quantifications of substrate phosphorylation are depicted below with the average and standard deviation plotted for each time point. **D)** Fluorescence imaging of nitrogen starved *S. cerevisiae* cells expressing neonGreen-tagged wild type Atg13 or the Atg13$^{S/T>A}$ mutant in the presence of either wild type Atg1 or catalytically inactive Atg1 (Atg1^{D211A}). **E)** Fluorescence imaging of exponentially growing (YPD) or nitrogen starved (SD-N) *S. cerevisiae* cells expressing either Atg13-neonGreen or neonGreen-Atg17. Atg13-neonGreen was imaged in either wild type or *atg11Δ*, *atg17Δ* or *atg11Δ atg17Δ* deletion backgrounds and neonGreen-Atg17 was imaged in either wild type or *atg11Δ*, *atg13Δ* or *atg11Δ atg13Δ* deletion backgrounds. **F)** Fluorescence imaging of exponentially growing (YPD) or nitrogen starved (SD-N) *S. cerevisiae* cells expressing either neonGreen-Atg17 or Atg1-neonGreen in the presence of wild type Atg13 (WT) or the Atg13$^{S/T>A}$ mutant. (**D-F**) Maximum intensity z-projections of deconvolved images are shown. Scale bar: 5 μm. **G)** Western blot quantification of the Cvt assay shown in Figure 3L (n=3).

Supplementary Figure 5. Atg8 lipidation is inhibited by Atg1 dependent E3 phosphorylation.

Related to Figure 4

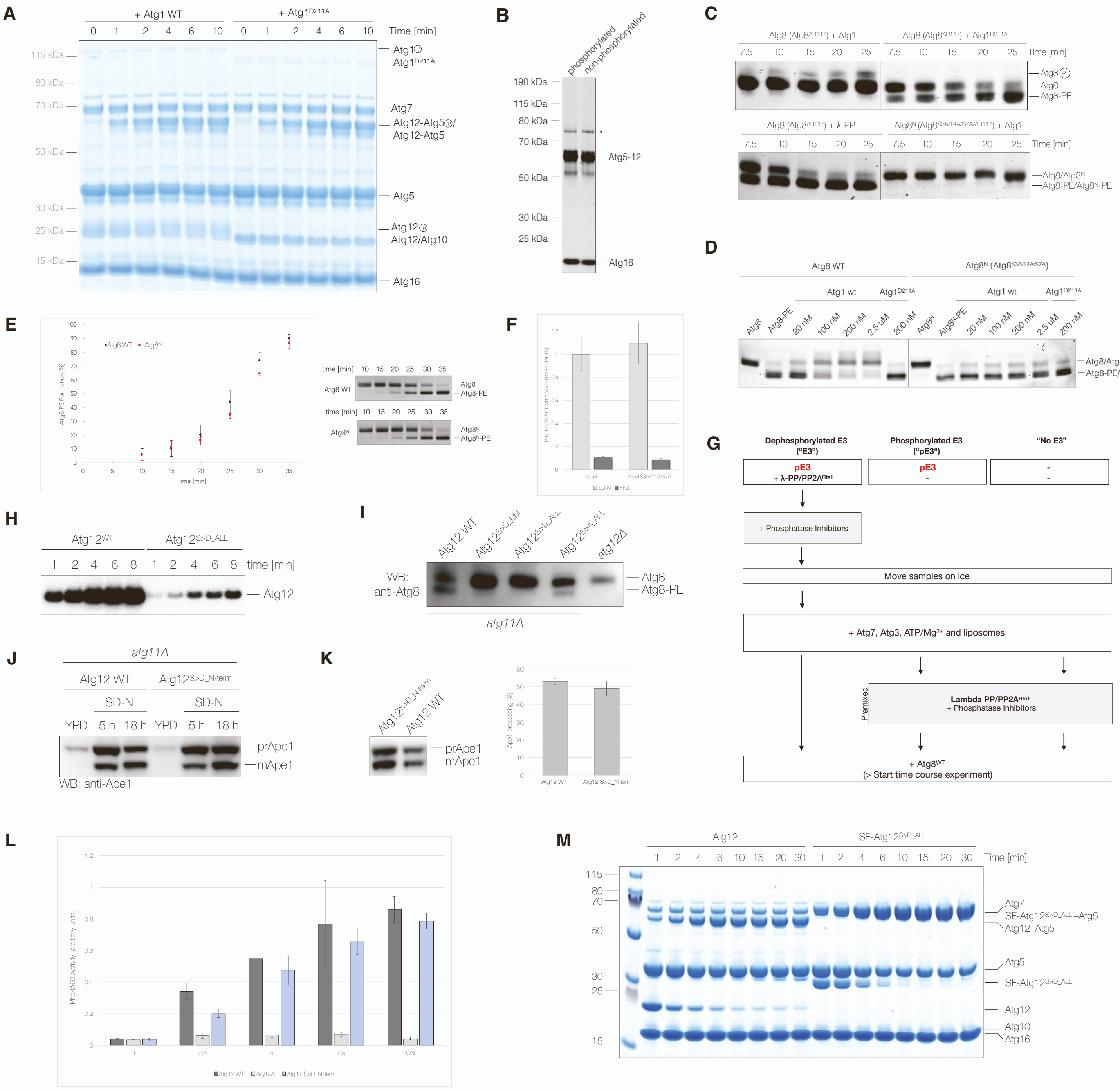

Supplementary Figure 5. Atg8 lipidation is inhibited by Atg1 dependent E3 phosphorylation.
A) Atg7, Atg10, Atg12 and the Atg5-Atg16 complex were individually incubated with either wild type Atg1 or catalytically inactive Atg1 (Atg1^{D211A}). Proteins were combined to initiate Atg12 conjugation to Atg5. Samples were taken at the indicated time points and analyzed by SDS-PAGE and Coomassie staining. **B)** SDS-PAGE comparison of Atg1 phosphorylated and non-phosphorylated Atg12–Atg5-Atg16 after anion exchange and size exclusion chromatography. Asterisk indicates co-purifying insect cell protein. **C**) Lipidation of Atg8 (Atg8$^{\Delta R117}$) and Atg8^{N} (Atg8$^{S3A/T4A/S7A/\Delta R117}$) are inhibited in the presence of Atg1 kinase activity. Full gel images of cropped images depicted in Figure 4B with neighbouring controls to confirm the inhibitory effect of Atg1 kinase activity on Atg8 and Atg8^{N} lipidation. **D)** Atg8 and Atg8^{N} were lipidated before incubating the reactions with increasing amounts of wild type Atg1 or catalytically inactive Atg1^{D211A}. The electrophoretic mobility shift of Atg8 and Atg8^{N} was monitored by Urea-SDS-PAGE and Sypro Ruby staining. **E)** *In vitro* lipidation kinetics of wild type Atg8 and Atg8^{N}. Samples were taken at the indicated time points and analyzed by Urea-SDS-PAGE and Sypro Ruby staining. The average Atg8-PE formation and standard deviation is plotted (n=3). **F)** The Pho8Δ60 assay was used to quantify bulk autophagy in *atg8Δ* strains or in cells expressing wild type Atg8 or the N-terminal alanine mutant Atg8^{N}. Cells were either exponentially grown in YPD medium or starved for 4 hours in nitrogen starvation medium (SD-N). Pho8Δ60 activity was measured in three independent experiments as described in 'Materials and Methods' and plotted as relative Pho8Δ60 activity with standard deviation. **G**) Schematic overview of the set up used to monitor Atg8 lipidation in the presence of either Atg1 phosphorylated or dephosphorylated E3 (pE3 and E3 respectively). Firstly, the E3 (Atg12–Atg5-Atg16-FLAG) was phosphorylated by Atg1 and subjected to a FLAG affinity purification and anion exchange chromatography step in order to minimize the Atg1 levels in the resultant pE3 preparation. To ensure that differences in Atg8 lipidation are not due to residual Atg1 kinase activity in the pE3 preparation the pE3 was dephosphorylated using lambda protein phosphatase (λ-PP) and recombinant PP2A^{Rts1} to act as the non-phosphorylated E3 control. The results and quantifications of the lipidation reactions are shown in Figure 4C. Liposomes used contained 25% PE, 45% PC, 5% PS and 25% PI. **H**) Wild type Atg12 and the phosphomimicking mutant, Atg12$^{S>D_ALL}$, were phosphorylated by Atg1 in a time dependent manner. Phosphorylation was analyzed by SDS-PAGE and auto-radiography. **I**) Atg8 lipidation was monitored in *atg11Δ* cells expressing either wild type Atg12 or the Atg12 mutants Atg12$^{S>D_ALL}$, Atg12$^{S>D_Ubl}$ or Atg12$^{S>A_ALL}$. Cells deleted for *ATG12* (*atg12Δ*) were used as a negative control. Cells were nitrogen starved for 18 hours and Atg8-PE formation was monitored by Western blotting using an anti-Atg8 antibody. **J**) Bulk autophagy was monitored *in vivo* using the Ape1 processing assay. Bulk autophagy dependent maturation of precursor Ape1 (prApe1) to its mature form (mApe1) was compared in *atg11Δ* cells expressing either wild type Atg12 or the phosphomimicking Atg12$^{S>D_N\text{-}term}$ mutant. Cells were either exponentially grown in YPD medium or nitrogen starved for 5 or 18 hours. Ape1 processing was analyzed by Western blotting using an anti-Ape1 antibody. **K**) The cytoplasm-to-vacuole targeting (Cvt) pathway was monitored in *S. cerevisiae* cells expressing either wild type Atg12 or the phosphomimicking Atg12$^{S>D_N\text{-}term}$ mutant. Cells were grown exponentially in nutrient-rich YPD medium and Ape1 processing was monitored by Western blotting. The conversion of precursor Ape1 (prApe1) to its mature form (mApe1) was quantified in the right panel (n=3). **L**) The Pho8Δ60 assay was used to quantify bulk autophagy in *atg12Δ* strains or in cells expressing wild type Atg12 or the phosphomimicking Atg12$^{S>D_N\text{-}term}$ mutant. Cells were either exponentially grown in YPD medium or nitrogen starved for 2.5, 5, 7.5 or 18 hours. Alkaline phosphatase activity was measured (n=3) and plotted as relative Pho8Δ60 activity with standard deviation. **M**) E3 complex formation was compared between wild type Atg12 and the phosphomimicking Atg12$^{S>D_ALL}$ mutant which has all detected Atg1 dependent *in vitro* phosphorylation sites mutated to aspartate. E3 complex formation was monitored in a time dependent manner after adding Atg12 or StrepII2x-FLAG-tagged Atg12$^{S>D_ALL}$ (SF-Atg12) to Atg5-Atg16, Atg10, Atg7 and ATP/Mg^{2+} containing reactions. Time points were analysed by SDS-PAGE and Coomassie staining.

Supplementary Figure 6. Atg8 lipidation is inhibited by Atg1 mediated phosphorylation of Atg3.
Related to Figure 5

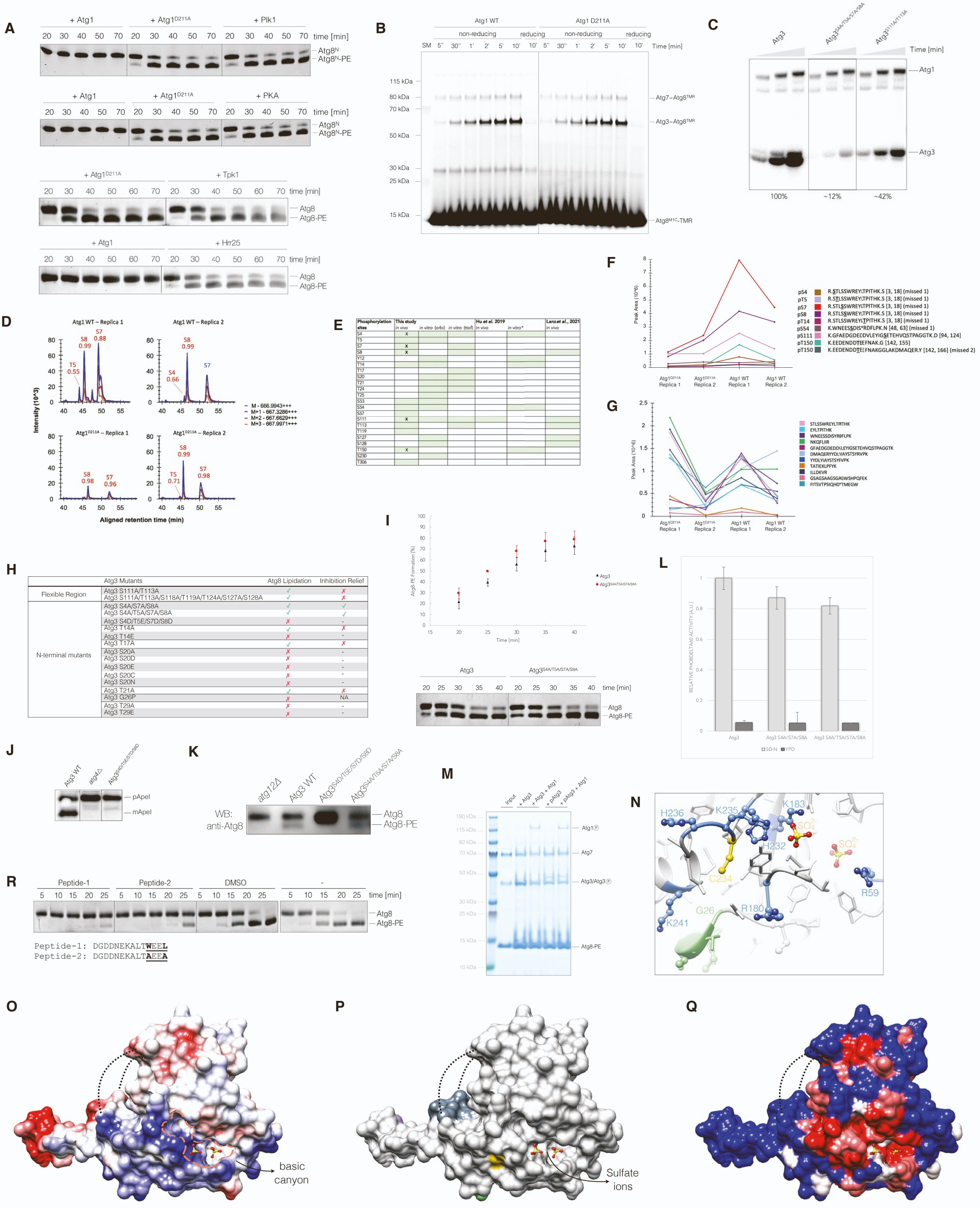

Supplementary Figure 6. Atg8 lipidation is inhibited by Atg1 mediated phosphorylation of Atg3. **A)** Atg3 and Atg7 were incubated with Atg1, Atg1^{D211A}, PKA, Polo-like kinase 1 (Plk1), Tpk1 or Hrr25 in the presence of ATP/Mg^{2+}, and Atg8 lipidation reactions were started by addition of liposomes and Atg8^{N} (Atg8$^{S3A/T4A/S7A/\Delta R117}$). Samples were taken at the indicated time points, and Atg8 lipidation was analyzed by Urea-SDS-PAGE and Sypro Ruby staining. **B)** Atg3 charging with fluorescently labeled Atg8^{M1C}-TMR was studied in the presence of wild type Atg1 or catalytically inactive Atg1^{D211A}. Samples were taken at the indicated time points, and Atg3 charging with Atg8^{M1C}-TMR was analyzed by SDS-PAGE under non-reducing conditions (- DTT). A reduced sample was included as control (+ DTT). SM: size marker. **C)** Atg1 *in vitro* kinase assays using either wild type Atg3, Atg3$^{S4A/T5A/S7A/S8A}$ or Atg3$^{S111A/T113A}$ as substrates. Phosphorylation was monitored in a time dependent manner and analyzed by SDS-PAGE and autoradiography. The rate difference is indicated below as percentage of wild type Atg3 phosphorylation. **D**) Quantitative mass spectrometry analysis of Atg3 derived phosphopeptides enriched after trypsin digestion of endogenous Atg3 purified from *S. cerevisiae* cells expressing either wild type Atg1 (Atg1 WT) or catalytically inactive Atg1 (Atg1^{D211A}). The extracted ion chromatography from MS1 scans for the first 4 isotopologues of the singly phosphorylated tryptic peptide STLSSWREYLTPITHK corresponding to the N-terminal phosphorylation sites of interest are shown. A number of positional isomers of phosphopeptides are observed separated in the chromatographic dimension. The chromatographic peaks are annotated with the amino acid position of the phosphorylation site, either where the MS2 spectrum was acquired in that sample (red font - including phosphosite localization probability), or by alignment from another sample (blue font). **E**) *S. cerevisiae* Atg3 *in vitro* and *in vivo* phosphorylation sites. The results from this study are compared to previously published data (Hu et al., 2019; Lanz et al., 2021). *In vivo* Atg1 dependent phosphorylation sites are highlighted by a cross. The signal intensity for all other *in vivo* phosphorylation sites was too low to accurately determine their Atg1 dependency. **F**) Relative quantification using MS1 extracted ion chromatogram peak areas for all Atg3 phosphopeptides enriched after trypsin digestion of endogenous Atg3 purified from *S. cerevisiae* cells expressing either wild type Atg1 or catalytically inactive Atg1^{D211A} analysed by quantitative mass spectrometry. **G**) Relative quantification using MS1 extracted ion chromatogram peak areas for all Atg3 non-phosphorylated peptides after trypsin digestion and phospho-enrichment of endogenous Atg3 purified from *S. cerevisiae* cells expressing either wild type Atg1 or catalytically inactive Atg1^{D211A} analysed by quantitative mass spectrometry. **H)** Overview of all Atg3 mutants tested in this study. Atg3 mutants were first tested in basic Atg8 lipidation assays to study the impact of the mutation(s) on basal catalytic activity. Only Atg3 mutants with uncompromised catalytic activity were tested in Atg1 containing Atg8 lipidation assays to study inhibition relief. Atg3 mutants which (partially) rescued Atg1 dependent inhibition mapped to the Atg3 N-terminus. **I)** Atg8 lipidation was compared in the presence of wild type Atg3 and the Atg3$^{S4A/T5A/S7A/S8A}$ mutant. Samples were taken at the indicated time points, and Atg8 lipidation was analyzed by Urea-SDS-PAGE and Sypro Ruby staining (bottom). Atg8 lipidation was quantified (n=3) and plotted as percentage (%) of total Atg8 levels (top graph). **J**) The selective Cvt pathway was monitored in *S. cerevisiae* cells expressing either wild type Atg3 or a phosphomimicking Atg3 mutant (Atg3$^{S4D/T5E/S7D/S8D}$). Cells were exponentially grown in YPD medium and Ape1 processing was monitored by Western blotting using an anti-Ape1 antibody. An *ATG4* deletion strain (*atg4Δ*) was included as a negative control. **K**) Atg8 lipidation was monitored in *S. cerevisiae* cells expressing either wild type Atg3 or the Atg3$^{S4D/T5E/S7D/S8D}$ or Atg3$^{S4A/T5A/S7A/S8A}$ mutants. Cells deleted for *ATG12* (*atg12Δ*) were used as negative control. Cells were nitrogen starved for 18 hours and Atg8-PE formation was monitored by Urea-SDS-PAGE and Western blotting using an anti-Atg8 antibody. **L**) The Pho8Δ60 assay was used to quantify bulk autophagy in cells expressing either wild type Atg3 or the Atg3$^{S4A/S7A/S8A}$ and Atg3$^{S4A/T5A/S7A/S8A}$ mutants. Cells deleted for *ATG3* (*atg3Δ*) were used as negative control. Cells were grown in either nutrient-rich YPD medium or nitrogen starved for 4 hours in starvation medium (SD-N). Alkaline phosphatase activity was measured (n=3) and plotted as relative Pho8Δ60 activity with standard deviation. **M)** Atg3 and pre-phosphorylated Atg3 were incubated with or without Atg1 and added to Atg8-PE containing liposomes. Liposomes were pelleted, washed and the pellets analyzed by SDS-PAGE. **N)** Close up view of the *S. cerevisiae* (*Sc*) Atg3 active site. Ribbon view of the *Sc*Atg3 crystal structure (PDB: 2DYT) highlighting the active site cysteine (C234) and the two sulfate ions (SO_4^{2-}) in the

neighbouring basic canyon. The N-terminal extension is depicted in green with the putative hinge residue glycine 26 (G26) highlighted. **O**) Surface view depicting the electrostatic (Coulomb) potential of *Sc*Atg3. Red indicates a negative and blue a positive potential. The basic canyon adjacent to the active site and the two bound sulfate ions (SO_4^{2-}) are indicated. **P**) Surface representation of Atg3 with domain color coding as shown in Figure 5D. The two sulfate ions occupy a cleft adjacent to the active site (in yellow). **Q**) Surface view of *Sc*Atg3 illustrating Atg3 sequence conservation. Red corresponds to high and blue to low sequence conservation. **R**) Two negatively charged peptides derived from the Atg19 C-terminus (300 μM) were added to Atg8 lipidation reactions. Both wild type and AIM mutant peptides (Peptide 1 and 2 respectively) were tested. Samples were taken at the indicated time points, and Atg8 lipidation was analyzed by Urea-SDS-PAGE and Sypro Ruby staining.

SUPPLEMENTAL INFORMATION

Supplemental Table 2. Yeast strains used in this study.

Related to STAR Methods

Strain Name	Genotype
yAS_602	*atg1Δ::*Atg1-neonGreen:natMX6
yAS_610	*atg1Δ::*Atg1-neonGreen:natMX6, *atg11Δ::URA3*
yAS_603	*atg1Δ::*Atg1^{D211A}-neonGreen:natMX6
yASC_799	*atg1Δ::*Atg1-neonGreen:natMX6, *atg13Δ::*Atg13$^{S/T>A}$:kanMX4
yAS_842	*atg1Δ::*Atg1$^{\Delta AIM/S>A}$-neonGreen:natMX6, *atg11Δ::URA3*
yAS_476*	*atg3Δ::*Atg3-SF:kanMX4
yAS_538*	*atg3Δ::*Atg3-SF:kanMX4, *atg1Δ::*Atg1^{D211A}:natMX6
yAS_293	*atg4Δ::natMX6*
yAS_233	*atg12Δ::*SF-Atg12:kanMX4
yASC_841	*atg12Δ::*SF-Atg12:kanMX4, *atg1Δ::*Atg1^{D211A}:natMX6
yASC_812	*ATG12:kanMX4*
yASC_761	*ATG12:kanMX4*, *atg11Δ::natMX6*
yASC_809	*atg12Δ::*Atg12$^{S>D_ALL}$:kanMX4
yASC_765b	*atg12Δ::*Atg12$^{S>D_ALL}$:kanMX4, *atg11Δ::natMX6*
yASC_810	*atg12Δ::*Atg12$^{S>A_ALL}$:kanMX4
yASC_755	*atg12Δ::*Atg12$^{S>A_ALL}$:kanMX4, *atg11Δ::natMX6*
yASC_811	*atg12Δ::*Atg12$^{S>D_Ubl}$:kanMX4
yASC_765c	*atg12Δ::*Atg12$^{S>D_Ubl}$:kanMX4, *atg11Δ::natMX6*
yASC_781	*atg12Δ::*Atg12$^{S>D_N\text{-}term}$:kanMX4
yASC_844	*atg12Δ::*Atg12$^{S>D_N\text{-}term}$:kanMX4, *atg11Δ::natMX6*
yAS_141	*atg12Δ::URA3*, *atg11Δ::natMX6*
yAS_223	*atg13Δ::*Atg13-neonGreen:kanMX4
yAS_266	*atg13Δ::*Atg13-neonGreen:kanMX4, *atg1Δ::*Atg1^{D211A}:natMX6
yAS_633	*atg13Δ::*Atg13-neonGreen:kanMX4, *atg9Δ::bleMX4*
yAS_634	*atg13Δ::*Atg13-neonGreen:kanMX4, *atg9Δ::bleMX4*, *atg1Δ::*Atg1^{D211A}:natMX6
yAS_554	*atg13Δ::*Atg13-neonGreen:kanMX4, *atg11Δ::URA3*, *atg1Δ::*Atg1^{D211A}:natMX6
yAS_621	*atg13Δ::*Atg13-neonGreen:kanMX4, *atg11Δ::URA3*
yASC_832	*atg13Δ::*Atg13-neonGreen:kanMX4, *atg17Δ::URA3*
yASC_820	*atg13Δ::*Atg13-neonGreen:kanMX4, *atg17Δ::URA3*, *atg11Δ::natMX6*
yAS_613	*atg13Δ::*Atg13-neonGreen:kanMX4, *atg8Δ::URA3*
yAS_614	*atg13Δ::*Atg13-neonGreen:kanMX4, *atg8Δ::URA3*, *atg1Δ::*Atg1^{D211A}:natMX6
yASC_784/8	*atg13Δ::*Atg13 $^{S/T>A}$:kanMX4
yASC_785	*atg13Δ::*Atg13 $^{S/T>A}$:kanMX4, *atg11Δ::natMX6*
yASC_830	*atg13Δ::*neonGreen-Atg13 $^{S/T>A}$:kanMX4
yASC_827	*atg13Δ::*neonGreen-Atg13 $^{S/T>A}$:kanMX4, *atg1Δ::*Atg1^{D211A}:natMX6
yAS_699	*atg17Δ::*neonGreen-Atg17:natMX6
yAS_713	*atg17Δ::*neonGreen-Atg17:natMX6, *atg1Δ::*Atg1^{D211A}:kanMX4
yASC_800	*atg17Δ::*neonGreen-Atg17:natMX6, *atg13Δ::*Atg13$^{S/T>A}$:kanMX4
yAS_721	*atg17Δ::*neonGreen-Atg17:natMX6, *atg11Δ::natMX6*
yASC_828	*atg17Δ::*neonGreen-Atg17:natMX6, *atg13Δ::URA3*

yASC_823	*atg17Δ::*neonGreen-Atg17:natMX6, *atg11Δ::natMX6*, *atg13Δ::URA3*
yAS_612	*atg29Δ::*Atg29-EGFP:His3MX6, *atg1Δ::*Atg1^{D211A}:natMX6
yAS_630	*atg31Δ::*Atg31-EGFP:His3MX6, *atg1Δ::*Atg1^{D211A}:natMX6
yAS_484	*pho8Δ*::Pho8Δ60:His3MX6, *pho13Δ::natMX6*, Atg1:kanMX4
yAS_477	*pho8Δ*::Pho8Δ60:His3MX6, *pho13Δ::natMX6*, *atg1Δ*::Atg1$^{S>A}$:kanMX4
yAS_542	*pho8Δ*::Pho8Δ60:His3MX6, *pho13Δ::natMX6*, *atg1Δ*::Atg1$^{\Delta AIM}$:kanMX4
yAS_555	*pho8Δ*::Pho8Δ60:His3MX6, *pho13Δ::natMX6*, *atg1Δ*::Atg1$^{\Delta AIM/S>A}$:kanMX4
yAS_316	*pho8Δ*::Pho8Δ60:His3MX6, *pho13Δ::natMX6*, *atg1Δ::URA3*
yAS_479	*pho8Δ*::Pho8Δ60:His3MX6, *pho13Δ::natMX6*, *Atg3:kanMX4*
yAS_473	*pho8Δ*::Pho8Δ60:His3MX6, *pho13Δ::natMX6*, *atg3Δ::*Atg3$^{S4A/S7A/S8A}$:kanMX4
yAS_472	*pho8Δ*::Pho8Δ60:His3MX6, *pho13Δ::natMX6*, *atg3Δ::*Atg3$^{S4A/T5A/S7A/S8A}$:kanMX4
yAS_474	*pho8Δ*::Pho8Δ60:His3MX6, *pho13Δ::natMX6*, *atg3Δ::*Atg3$^{S4D/T5E/S7D/S8D}$:kanMX4
yASC_779	*pho8Δ*::Pho8Δ60:His3MX6, *pho13Δ::natMX6*, *atg3Δ::*Atg3$^{S4A/T5A/S7A/S8A}$:bleMX4, *atg12Δ::*Atg12$^{S>A_ALL}$:kanMX4
yASC_780	*pho8Δ*::Pho8Δ60:His3MX6, *pho13Δ::natMX6*, *atg3Δ::*Atg3$^{S4A/T5A/S7A/S8A}$:bleMX4, *atg12Δ::*Atg12$^{S>A_Ubl}$:kanMX4
yAS_313	*pho8Δ*::Pho8Δ60:His3MX6, *pho13Δ::natMX6*, *atg3Δ::*URA3
yAS_486	*pho8Δ*::Pho8Δ60:His3MX6, *pho13Δ::natMX6*, Atg12:kanMX4
yASC_757	*pho8Δ*::Pho8Δ60:His3MX6, *pho13Δ::natMX6*, *atg12Δ::*Atg12$^{S>A_ALL}$:kanMX4
yASC_768	*pho8Δ*::Pho8Δ60:His3MX6, *pho13Δ::natMX6*, *atg12Δ::*Atg12$^{S>D_ALL}$:kanMX4
yASC_764	*pho8Δ*::Pho8Δ60:His3MX6, *pho13Δ::natMX6*, *atg12Δ::*Atg12$^{S>D_Ubl}$:kanMX4
yASC_845	*pho8Δ*::Pho8Δ60:His3MX6, *pho13Δ::natMX6*, *atg12Δ::*Atg12$^{S>D_N\text{-}term}$:kanMX4
yASC_763	*pho8Δ*::Pho8Δ60:His3MX6, *pho13Δ::natMX6*, *atg12Δ*::URA3
yASC_783	*pho8Δ*::Pho8Δ60:His3MX6, *pho13Δ::natMX6*, Atg13 $^{S/T>A}$:kanMX4

The Atg29-GFP:His3MX6 and Atg31-GFP:His3MX6 strains were taken from the GFP collection (Huh et al., 2003).
Atg1$^{\Delta AIM}$: Atg1^{Y429A_V432A}; Atg1$^{S>A}$: Atg1^{S418A_S421A_S424A}; Atg1$^{\Delta AIM/S>A}$: Atg1^{S418A_S421A_S424A_Y429A_V432A}
* Atg3-SF refers to an Atg3 mutant which has the StrepII2x-FLAG-tag inserted between amino acids 266 and 268 replacing amino acid 267.

SUPPLEMENTAL INFORMATION

Supplemental Table 3. Plasmids used in this study.

Related to STAR Methods

Plasmids Used for Yeast Strain Construction

Gene Construct	Plasmid Name
Atg1:kanMX4	pAS_374
Atg1^{D211A}:kanMX4	pAS_393
Atg1^{D211A}:natMX6	pAS_580
Atg1::URA3	pAS_385
Atg1$^{S>A}$:kanMX4	pAS_843
Atg1$^{\Delta AIM}$:kanMX4	pAS_842
Atg1$^{\Delta AIM/S>A}$:kanMX4	pAS_972
Atg11::natMX6	pAS_141
Atg11::URA3	pAS_590
Atg13::URA3	pAS_234
Atg1^{D211A}-neonGreen:natMX6	pAS_1004*
Atg13$^{S/T>A}$:kanMX4	pASC_316
Atg13$^{S/T>A}$-neonGreen:kanMX4	pASC_381
Atg13-neonGreen:kanMX4	pAS_454
Atg12::URA3	pAS_341
Atg12:kanMX4	pAS_342
SF-Atg12:kanMX4	pAS_357
Atg12$^{S>D_N\text{-}term}$:kanMX4	pASC_304
Atg12$^{S>D_Ubl}$:kanMX4	pASC_305
Atg12$^{S>D_ALL}$:kanMX4	pASC_220
Atg12$^{S>A_ALL}$:kanMX4	pASC_221
Atg12$^{S>A_Ubl}$:kanMX4	pASC_306
Atg17::URA3	pAS_281
neonGreen-Atg17:natMX6	pAS_1065
Atg3^{WT}:kanMX4	pAS_376
Atg3^{SF}:kanMX4	pAS_810
Atg3$^{S4A/S7A/S8A}$:kanMX4	pAS_847
Atg3$^{S4A/T5A/S7A/S8A}$:kanMX4	pAS_846
Atg3$^{S4A/T5A/S7A/S8A}$:bleMX4	pAS_868
Atg3$^{S4D/T5E/S7D/S8D}$:kanMX4	pAS_835
Atg9::bleMX4	pAS_1021

*pAS_1004 was used to create both yAS_602 (NdeI/NotI) and yAS_603 (NheI/NotI) by using different restriction enzymes for linearization; Atg12$^{S>A_ALL}$: S13A, S16A, S17A, S30A, S38A, S39A, S55A, S61A, S64A, S72A, S73A, S74A, S113A, S120A, S127A, S153A; Atg12$^{S>D_ALL}$: S13D, S16D, S17D, S30D, S38D, S39D, S55D, S61D, S64D, S72D, S73D, S74D, S113D, S120D, S127D, S153D; Atg12$^{S>D_Ubl}$: S113D, S120D, S127D, S153D; Atg12$^{S>D_N\text{-}term}$: S13D, S16D, S17D, S30D, S38D, S39D, S55D, S61D, S64D, S72D, S73D, S74D; Atg13$^{S/T>A}$ mutants: T269A, S275A, S280A, S282A, S285A, S298A, T333A, S344A, S346A, S348A, T351A, S355A, S360A, S369A, S379A, S382, S384A, S386A, S390A, S392A, S393A, S404A, S406A, S407A, T418A, S419A, S422A, T423A, S424A, S427A, S428A, S429A, S437A, S438A, T441A, T442A, S449A, S454A, S461A, T479A, T483A, S484A, S494A, S496A, S506A, S511A, S515A, S517A and S519A; Atg1$^{\Delta AIM}$: Atg1^{Y429A_V432A}; Atg1$^{\Delta AIM/S>A}$: Atg1^{Y429A_V432A_S418A_S421A_S424A}; Atg1$^{S>A}$: Atg1^{S418A_S421A_S424A}.

Plasmids Used for Bacterial Expression

Constructs	Plasmid	Backbone
GST-Atg8$^{\Delta\text{R117}}$	pAS_053	pGEX-5X-1
SF-Atg8$^{\Delta\text{R117}}$	pAS_054	pET17b
SF-Atg8$^{\Delta\text{G116}/\Delta\text{R117}}$	pASC_260	pET17b
SF-tagged Atg8$^{\text{M1C}/\Delta\text{R117}}$	pAS_511	pET17b
SF-Atg8$^{\text{S3A/T4A/S7A}/\Delta\text{R117}}$	pAS_220	pET17b
Atg3-StrepII^{2x}	pAS_382	pET17b
Atg3$^{\text{S4A/S7A/S8A}}$-StrepII^{2x}	pAS_570	pET17b
Atg3$^{\text{S4A/T5A/S7A/S8A}}$-StrepII^{2x}	pAS_676	pET17b
Atg3$^{\text{S4D/T5E/S7D/S8D}}$-StrepII^{2x}	pAS_838	pET17b
Atg3$^{\text{S111A/T113A}}$-StrepII^{2x}	pAS_495	pET17b
Atg3$^{\text{S111A/T113A/S118A/T119A/T124A/S127A/S128A}}$-StrepII^{2x}	pAS_585	pET17b
Atg3$^{\text{T14A}}$-StrepII^{2x}	pAS_642	pET17b
Atg3$^{\text{T14E}}$-StrepII^{2x}	pAS_656	pET17b
Atg3$^{\text{T17A}}$-StrepII^{2x}	pAS_648	pET17b
Atg3$^{\text{S20A}}$-StrepII^{2x}	pAS_641	pET17b
Atg3$^{\text{S20D}}$-StrepII^{2x}	pAS_655	pET17b
Atg3$^{\text{S20E}}$-StrepII^{2x}	pAS_657	pET17b
Atg3$^{\text{S20C}}$-StrepII^{2x}	pAS_774	pET17b
Atg3$^{\text{S20N}}$-StrepII^{2x}	pAS_658	pET17b
Atg3$^{\text{T21A}}$-StrepII^{2x}	pAS_647	pET17b
Atg3$^{\text{G26P}}$-StrepII^{2x}	pAS_833	pET17b
Atg3$^{\text{T29A}}$-StrepII^{2x}	pAS_639	pET17b
Atg3$^{\text{T29E}}$-StrepII^{2x}	pAS_654	pET17b
Atg10-StrepII^{2x}	pAS_221	pET17b
Atg16-Atg5-StrepII2x	pAS_505	pETDuet-1
Atg16	pAS_515	pETDuet-1
Atg16-FLAG	pAS_1071	pETDuet-1
SH-SUMO*-Atg1$^{\Delta\text{KD}_\Delta\text{AIM/S>A}}$	pASC_250	pET17b
SH-SUMO*-Atg1$^{\Delta\text{KD}_\Delta\text{MIT}_\Delta\text{AIM/S>A}}$	pASC_251	pET17b
SH-SUMO*-Atg1^{MIT}	pASC_107	pET17b
SH-SUMO*-Atg13$^{\Delta\text{HORMA}_\Delta\text{C}}$	pASC_216	pET17b
SH-SUMO*-Atg13$^{\Delta\text{HORMA}_\Delta\text{C}_\text{TOR}_\text{S>A}}$	pASC_252	pET17b
SH-SUMO*-Atg13$^{\Delta\text{HORMA}_\Delta\text{C}_\text{pS>A}}$	pASC_282	pET17b
SH-SUMO*-Atg13$^{\Delta\text{HORMA}_\Delta\text{C}_\text{S/T>A}}$	pASC_295	pET17b
SH-SUMO*-Sic1	pASC_247	pET17b
SH-SUMO*-Tpk1	pASC_279	pET17b

SF-tag: StrepII^{2x}-FLAG-tag; SH-SUMO*-tag: His$_6$-StrepII^{2x}-SUMO*-tag; KD: kinase domain; AIM: Atg8-interacting motif; Atg13$^{\Delta\text{HORMA}_\Delta\text{C}}$: Atg13$^{269\text{-}521}$; Mutations in Atg13$^{\Delta\text{HORMA}_\Delta\text{C}_\text{pS>A}}$: S280A, S282A, S285A, S344A, S346A, S355A, S360A, S369A, S382, S384A and S386A. Mutations in Atg13$^{\Delta\text{HORMA}_\Delta\text{C}_\text{S/T>A}}$ and Atg13$^{\text{S/T>A}}$ mutants: T269A, S275A, S280A, S282A, S285A, S298A, T333A, S344A, S346A, S348A, T351A, S355A, S360A, S369A, S379A, S382, S384A, S386A, S390A, S392A, S393A, S404A, S406A, S407A, T418A, S419A, S422A, T423A, S424A, S427A, S428A, S429A, S437A, S438A, T441A, T442A, S449A, S454A, S461A, T479A, T483A, S484A, S494A, S496A, S506A, S511A, S515A, S517A and S519A.

Plasmids Used for Baculovirus Generation and Insect Cell Expression

Expression Constructs	Plasmid	Backbone
SH-SUMO*Atg7	pAS_144	pFBDM
SF-Atg12	pAS_333	pFBDM
SF-Atg12$^{S>D_All}$	pASC_319	pFBDM
Atg7-Atg10-Atg12-Atg5-StrepII^{2x}	pAS_522	pFBDM
Atg7-Atg10-Atg12 $^{S>D\_Ubl}$-Atg5-StrepII^{2x}	pASC_364	pFBDM
Atg16-Atg5-StrepII^{2x}	pAS_179	pFBDM
Atg4-StrepII^{2x}	pAS_240	pFBDM
Atg4^{C147A}-StrepII^{2x}	pAS_1070	pFBDM
Atg9-StrepII^{2x}	pAS_673	pFBDM
StrepII^{2x}-Vps15^{G2A}-Vps34-Atg6-Atg14-Atg38	pAS_266	pFBDM
StrepII^{2x}-Vps15^{G2A}-Vps34-Atg6-Atg14-FLAG	pAS_262	pFBDM
Atg2-StrepII^{2x}-Atg18-FLAG	pAS_301	pFBDM
Atg2-StrepII^{2x}	pAS_389	pFBDM
Atg18-StrepII^{2x}	pAS_398	pFBDM
StrepII^{2x}-Atg1	pAS_082	pFBDM
StrepII^{2x}-Atg1^{D211A}	pAS_917	pFBDM
StrepII^{2x}-Atg1$^{\Delta AIM}$	pAS_918	pFBDM
StrepII^{2x}-Atg1$^{\Delta AIM/S>A}$	pAS_971	pFBDM
StrepII^{2x}-Atg1$^{\Delta AIM/S>A_D211A}$	pASC_357	pFBDM
StrepII^{2x}-Atg1$^{\Delta MIT_\Delta AIM}$	pASC_172	pFBDM
StrepII^{2x}-Atg1$^{\Delta MIT}$	pASC_173	pFBDM
SH-SUMO*-Atg13-FLAG	pAS_946	pFBDM
StrepII2x-Atg13-Myc	pASC_203	pFBDM
StrepII2x-Atg13$^{S/T>A}$-Myc	pASC_333	pFBDM
StrepII^{2x}-Atg1-Atg13	pAS_090	pFBDM
StrepII^{2x}-Atg1^{D211A}-Atg13	pAS_247	pFBDM
StrepII^{2x}-Atg1^{D211A}-Atg13$^{S/T>A}$	pASC_367	pFBDM
StrepII^{2x}-Atg1-Atg13$^{\Delta HORMA}$	pAS_936	pFBDM
StrepII^{2x}-Atg1-Atg13$^{TOR_S>A}$	pAS_913	pFBDM
SF-Atg17-Atg29-Atg31	pAS_455	pFBDM
SH-SUMO*-Hrr25	pASC_266	pFBDM
PP2A^{Rts1} (StrepII^{2x}-Rts1-Pph22-Tpd3)	pAS_689	pFBDM

SH-SUMO*-tag: His$_6$-StrepII^{2x}-SUMO*; Atg1$^{\Delta AIM}$: Atg1^{Y429A_V432A}; Atg1$^{\Delta AIM/S>A}$: Atg1^{Y429A_V432A_S418A_S421A_S424A}; Atg1$^{\Delta MIT}$: Atg1$^{1\text{-}624}$; Atg13$^{TOR_S>A}$: Atg13^{S379A_S428A_S429A}; Atg13$^{\Delta HORMA}$: Atg13$^{269\text{-}728}$; Atg12$^{S>D_ALL}$: S13D, S16D, S17D, S30D, S38D, S39D, S55D, S61D, S64D, S72D, S73D, S74D, S113D, S120D, S127D, S153D; Atg12$^{S>D_Ubl}$: S113D, S120D, S127D, S153D. Atg13$^{S/T>A}$: T269A, S275A, S280A, S282A, S285A, S298A, T333A, S344A, S346A, S348A, T351A, S355A, S360A, S369A, S379A, S382, S384A, S386A, S390A, S392A, S393A, S404A, S406A, S407A, T418A, S419A, S422A, T423A, S424A, S427A, S428A, S429A, S437A, S438A, T441A, T442A, S449A, S454A, S461A, T479A, T483A, S484A, S494A, S496A, S506A, S511A, S515A, S517A and S519A.
